# Anti-Contractile and Anti-Inflammatory Effects of Diacerein on Isolated Mouse Airways Smooth Muscle and Mouse Asthma Model

**DOI:** 10.3389/fphar.2020.560361

**Published:** 2020-09-04

**Authors:** Shunbo Shi, Lu Xue, Shuhui Han, Haiting Qiu, Yongbo Peng, Ping Zhao, Qing-Hua Liu, Jinhua Shen

**Affiliations:** Institute for Medical Biology and Hubei Provincial Key Laboratory for Protection and Application of Special Plants in Wuling Area of China, College of Life Sciences, South-Central University for Nationalities, Wuhan, China

**Keywords:** asthma, diacerein, ion channels, calcium mobilization, inflammation

## Abstract

Characterized by abnormal smooth muscle contractility and airway inflammation, asthma is one of the most common airway diseases worldwide. Diacerein is a well-known anti-inflammatory drug, widely used in osteoarthritis. In current study, the innovative usage of diacerein in anti-contractile and anti-inflammatory treatment of asthma was studied. *In vitro* experiments including tension measurement and patch-clamp technique and *in vivo* experiments including establishment of mice model and measurement of respiratory resistance were applied to explore the role of diacerein in asthma. It turned out that agonist-precontracted mouse airway smooth muscle could be relaxed by diacerein *via* intracellular and extracellular calcium mobilization which was mediated by switched voltage-dependent L-type Ca^2+^ channels, non-selective cation channels, large-conductance Ca^2+^-activated K^+^ channel, and Na^+^/Ca^2+^ exchangers. Furthermore, diacerein could relieve bronchospasm and control airway inflammation in asthmatic mice *via* reduction of several inflammatory factors. Our studies elucidated the potential therapeutic property of diacerein in asthma treatment and the possible underlying mechanism. It also confirmed that new uses for already-approved drugs could be an important form of innovation.

## Introduction

Asthma is a heterogeneous and chronic inflammatory disease mainly characterized by reversible bronchial obstruction, expiratory airflow limitation, airway hyperresponsiveness, remodeling, and inflammation (Holgate, 2012; Wenzel, 2012; Nieto-Fontarigo et al., 2019). As a comprehensive syndrome of various phenotypes with defined clinical and physiological characteristics, asthma is a global public health issue, with the recent data from Global Initiative for Asthma suggesting that over 300 million individuals are suffering from this condition (Moorman et al., 2012; Beasley et al., 2015; Becker and Abrams, 2017). Increasing prevalence, reduced quality of life, and extra economic burden made asthma an expensive and challenging concern for individuals and whole society (Bahadori et al., 2009; Moorman et al., 2012; Chen et al., 2018).

For decades, great efforts have been made to prevent asthma and relieve symptoms (Adcock et al., 2008; Engelkes et al., 2015; Zhu et al., 2018). Plenty of asthma medications including β-agonists and inhaled corticosteroids have been designed to relax abnormal airway muscle contraction and minimize airway inflammatory reaction (Bleecker et al., 2016; Wenzel et al., 2016; Sobieraj et al., 2018a; Sobieraj et al., 2018b). However, as the mainstay of asthma therapy, the conventional use of bronchodilators or inhaled corticosteroids might be limited on partly severe asthma patients (Fajt and Wenzel, 2017). Furthermore, long-term usage of high-dose inhaled corticosteroids might increase the risk of pneumonia (McKeever et al., 2013), nontuberculous mycobacterial pulmonary disease (Brode et al., 2017), cataracts, osteoporosis in elderly patients (Heffler et al., 2018), and hypothalamic-pituitary-adrenal (HPA) axis suppression in children (Kapadia et al., 2016). The development of more effective drugs for asthma with less side effects are urgently needed (Fajt and Wenzel, 2017).

To solve the problem, a series of anti-inflammatory mediators with different pathogenic targets have been proposed to asthma treatment (Celotti and Laufer, 2001; Zhu et al., 2018). Besides well-known omalizumab (Strunk and Bloomberg, 2006), mepolizumab (Ortega et al., 2014) and benralizumab (Nair et al., 2017), tofacitinib, a Janus kinase-signal transducers and activators of transcription (JAK-STAT) signal inhibitor, has exhibited anti-inflammatory effects in murine asthma models and represent the potential to be an effective treatment for asthma (Younis et al., 2019). Accumulating evidence indicated that anti-inflammatory drugs could exert strong efficacy in asthma patients, which enlighten us that developing new uses of already-approved agents with anti-inflammatory properties might be a simple and effective way to pursue innovation of asthma drugs.

Diacerein (1,8-diacetoxy-9,10-dioxo-dihydroanthracene-3-carboxylic acid), which is an anthraquinone synthesized in 1980 that interferes with an inflammatory mediator, interleukin 1 (IL-1) has been proposed as a symptomatic slow-acting drug for joint diseases, such as osteoarthritis (OA) (Fidelix et al., 2014; Pavelka et al., 2016). The principal mechanism of diacerein is to reduce production of IL-1 converting enzyme then inhibit the activation of IL-1β by related downstream signaling (Martel-Pelletier and Pelletier, 2010). Due to its anti-inflammatory properties, diacerein was also considered to be a treatment option for type 2 diabetes (Zhang et al., 2017). Moreover, previous studies have revealed that rhein, an active metabolite of diacerein, could suppress human aortic smooth muscle cell proliferation (Heo et al., 2009) and decrease contractility of isolated colon and uterus from rat (Odenthal and Ziegler, 1988). However, the study of diacerein on asthma, which present typical inflammatory symptom, abnormal proliferative activity, and excessive contractility is still limited.

The aim of present study was to investigate the roles of diacerein in asthma treatments with a focus on anti-contractile and anti-inflammatory properties. *In vitro* experiments showed that diacerein exerted relaxant characteristic on agonist-precontracted airway smooth muscle in a concentration-dependent way. Meanwhile, intracellular and extracellular calcium mobilization played a pivotal role during diacerein-induced relaxation as a result of switched voltage-dependent L-type Ca^2+^ channels (VDLCCs), non-selective cation channels (NSCCs), large-conductance Ca^2+^-activated K^+^ channel (BK channel), and Na^+^/Ca^2+^ exchangers (NCX). Further *in vivo* experiments showed that diacerein could significantly relieve asthmatic symptoms including airway hyperresponsiveness, airway remodeling, inflammation, and mucus secretion. Our research provided primary evidence that diacerein might have potential therapeutic value for clinical treatment of asthma.

## Materials and Methods

### Reagents and Chemicals

Diacerein was purchased from GuoDa Drugstore (Shanghai, China) and dissolved in deionized distilled water for further use. Acetylcholine chloride (ACh) was purchased from Yuanye Bio-Technology Co., Ltd (Shanghai, China). Bovine serum albumin (BSA), cesium chloride (CsCl), collagenase H, dithiothreitol (DTT), gadolinium, MgATP, nifedipine, niflumic acid (NA), ovalbumin (OVA), papain, paxilline (PAX), pyrazole 3 (Pyr3), and tetraethylammonium chloride (TEA) were purchased from Sigma (St. Louis, MO, USA). TRIzol^®^ was purchased from Invitrogen (Carlsbad, CA, USA). RNA extraction kit and complementary DNA (cDNA) synthesis kit were purchased from Takara (Otsu, Japan). SYBR^®^ Green Realtime PCR Master mix was purchased from Toyobo (Osaka, Japan). All other chemicals were purchased from Sinopharm Chemical Reagent Co. (Shanghai, China).

### Animals

All the animal experiments were designed and performed as previously described with minor revision (Wen et al., 2020). Briefly, male BALB/c mice at 6–8 weeks of age were obtained from the Hubei Provincial Center for Disease Control and Prevention (Wuhan, China). The mice were housed in a specific pathogen-free (SPF)-grade laboratory under a 12 h light-dark cycle. All animal experiments were approved by the Animal Care and Ethics Committee of the South-Central University for Nationalities (Wuhan, China) and were performed under the supervision of the same institute (Wuhan, China).

### Establishment of Asthma and Diacerein-Treated Models

Six-week-old mice were randomly allocated to three groups thereafter named as control group, asthma group, and diacerein group, respectively. Asthma group and diacerein group were sensitized by intraperitoneally (IP) injecting 200 μl of 3 mg/ml OVA dissolved with 25 mg/ml aluminum hydroxide [Al(OH)_3_] at day 1. The second injection was performed at day 8 with 15 mg/ml Al(OH)_3_. From day 15, asthma and diacerein group were stimulated (once per day) by intranasal instillation of OVA (3 mg/ml, 50 μl) diluted with normal saline solution (NSS). Meanwhile, diacerein group was also gavaged daily with diacerein (50, 100, 200 mg/kg, respectively). Control group was parallelly treated with NSS. A week after stimulation, mice were euthanized, then the tracheal and lung were collected and photographed for further experiments.

### Measurement of Mouse Airway Smooth Muscle Tension

The tension of mouse tracheal rings (mTRs) was measured in an isometrically manner as previously described (Wen et al., 2020). mTRs (5~7 mm) were cut from euthanized mice and quickly transferred to ice-cold physiological salts solution (PSS) (in mM: NaCl 135, MgCl_2_·6H_2_O 1, CaCl_2_ 2, glucose 10, HEPES 10, KCl 5, pH adjusted to 7.4 with 5 M NaOH) or Li-PSS (in mM: KCl 5, glucose 10, HEPES 10, LiCl 135, CaCl_2_ 2, MgCl_2_·6H_2_O 1, pH adjusted to 7.4 with Tris-base) or 0 Ca^2+^-PSS (in mM: KCl 5, glucose 10, HEPES 10, EGTA 0.5, MgCl_2_·6H_2_O 1, NaCl 135, pH adjusted to 7.4 with 5 M NaOH) according to experimental design. Each mTR was suspended in a 10 ml organ bath filled with PSS/Li-PSS/Ca^2+^-free PSS bubbled continuously with 95% O_2_ and 5% CO_2_ at 37°C. After a 60 min equilibration, mTRs were given a successive stimulation with either high K^+^ (80 mM) or ACh (100 μM) for precontraction then the tension measurements were conducted and certain reagents were added according to experimental design.

### Isolation of Mouse Airway Smooth Muscle Cells

Single mouse airway smooth muscle cells (mASMCs) was isolated from freshly removed mouse tracheal as previously described with minor revision (Chen et al., 2019). Briefly, mouse tracheal muscles were isolated and collected in mASMC dissociation buffer (in mM: CaCl_2_ 0.1, NaHCO_3_ 25, KH_2_PO_4_, HEPES 10, KCl 5.2, MgCl_2_ 1.2, glucose 11, NaCl 120, pH adjusted to 7.0 with 5 M NaOH). Then the collected muscles were incubated in digest solution I (in mg/ml: BSA 1, papain 2, DTT 0.15) at 37°C for 23–25 min, following by transferring to digest solution II (in mg/ml: BSA 1, collagenase H 1) at 37°C for 6–8 min. The digested tissues were washed and gently pipetted with 1 mg/ml BSA to yield single mASMC for further experiments.

### Measurement of Channel Currents

The channel currents were measured using the whole-cell recording technique *via* an EPC-10 patch-clamp amplifier (HEKA, Lambrecht, Germany) as previously described with minor revision (Chen et al., 2019; Wen et al., 2020).

For the measurement of VDLCC currents, the pipette was filled with intracellular solution (in mM: MgCl_2_ 4, MgATP 4, EGTA 10, HEPES 10, CsCl 130, pH adjusted to 7.2 with CsOH). mASMCs were patched and held in the bath solution (in mM: HEPES 10, CsCl 6, glucose 11, TEA 10, NaCl 105, BaCl_2_ 27.5, pH adjusted to 7.4 with 5 M NaOH) at −70 mV. VDLCC currents were activated and measured under a stepped voltage ranged from −70 to +40 mV in 10 mV increments every 50 ms.

For the measurement of NSCC currents, the pipette was filled with intracellular solution (in mM: CaCl_2_ 1, MgCl_2_ 1.2, HEPES 10, EGTA 3, CsCl 18, caesium acetate 108, pH adjusted to 7.2 with Tris-base). mASMCs were patched and held in the bath solution (in mM: CaCl_2_ 1.5, HEPES 10, glucose 11, NaCl 126, pH adjusted to 7.2 with 5M NaOH) at −60 mV. NSCC currents were recorded with a 500 ms ramp from −80 to +60 mV and the data at 70 mV was used to construct current-time curves.

For the measurement of K^+^ channel currents, the pipette was filled with intracellular solution (in mM: EGTA 10, MgCl_2_ 6.2, HEPES 10, NaCl 10, KCl 125, pH adjusted to 7.2 with KOH). mASMCs were patched and held in the bath solution (in mM: CaCl_2_ 5.4, MgCl_2_ 0.8, HEPES 10, NaCl 150, KCl 5.4, pH adjusted to 7.2 with KOH). K^+^ currents were recorded from a holding potential −80 to +80 mV in 10 mV increments.

### Measurement of Respiratory System Resistance

Respiratory system resistance (Rrs) was measured using a forced oscillation technique as previously described with some modifications (Chen et al., 2019). Briefly, mice were set under general anesthesia (intraperitoneal sodium pentobarbital 10 mg/kg), tracheostomized *via* an 18G metal cannula, placed in a flow-type body plethysmograph and connected with a flexiVent system (SCIREQ, Montreal, PQ, Canada). Each mouse was mechanically ventilated with a tidal volume of 10 ml/kg, 150 breaths/min, positive end expiratory pressure (PEEP) of 3 cm H_2_O. A dose-response curve was generated by administering aerosolized ACh for 150 s at increasing doses (3.125, 6.25, 12.5, 25, and 50 mg/ml).

### Histology Processing and Staining

Trachea and lung specimens isolated from different experimental groups were fixed in 4% paraformaldehyde and paraffin-embedded then sliced into 3 µm-thick sections. Before staining, the tissue was baked for 20–30 min, then deparaffinized with xylene and gradually rehydrated with 100% ethanol, 90% ethanol, 80% ethanol, 70% ethanol, and double distilled water. Histological staining was performed using hematoxylin and eosin (H&E) solution using standard histological protocols. For periodic acid-Schiff (PAS) staining, tissue sections were stained in 0.5% periodic acid for 5 min and Schiff’s Reagent for 15 min, respectively. After counterstaining with hematoxylin, sections were rinsed with water, dehydrated, and mounted. Stained sections were photographed and analyzed under a bright-field microscope.

### Reverse Transcription Quantitative Real-Time PCR

Total RNA was obtained from right lung of mice (asthma group, diacerein group, and control group) and cDNA was synthesized from 0.1 ng to 5 µg RNA according to manufacturer’s instructions. Real-time PCR and melting curve analysis were performed with SYBR^®^ Green Realtime PCR Master Mix using the Applied Biosystems 7500 Fast Real-Time PCR System^®^ (Foster City, CA, USA) under defaulted program. The messenger RNA (mRNA) expression levels of inflammatory factors were calculated relative to Actin (internal control) utilizing 2^−ΔΔCt^ method. The sequences of primers were shown as following:

**Table:** 

Primer name	Sequences	Product sizes (bp)
Actin-F	5’- AGAGGGAAATCGTGCGTGAC -3’	117
Actin-R	5’- CAATAGTGATGACCTGGCCGT -3’	
Muc5ac-F	5’- AGTCTCTCTCCGCTCCTCTCAAT -3’	176
Muc5ac-R	5’- CAGCCGACACCACCCTTTGATCT -3’	
IL-12b-F	5’- ACGGCCAGAGAAAAACTGAA -3’	198
IL-12b-R	5’- CTACCAAGGCACAGGGTCAT -3’	
IL-13-F	5’- CACACAAGACCAGACTCCCC -3’	268
IL-13-R	5’- CCAGGGATGGTCTCTCCTCA -3’	
IL-4-F	5’- AACGAAGAACACCACAGAGAGTG -3’	137
IL-4-R	5’- CGATGAATCCAGGCATCGAAAAG -3’	
TNF-F	5’- TGGAAGACTCCTCCCAGGTA -3’	255
TNF-R	5’- ACGGCATGGATCTCAAAGAC -3’	

### Statistical Analysis

All data were expressed as the means ± standard deviation (SD). For all analyses, the evaluations were carried out with student’s *t*-test or one-way ANOVA using Origin 8.0 software (OriginLab, Northampton, MA, USA). *p* < 0.05 was regarded as statistically significant.

## Results

### Diacerein Relaxed High K^+^-Induced Precontraction in a Dose-Dependent Manner

**Figure 1:**
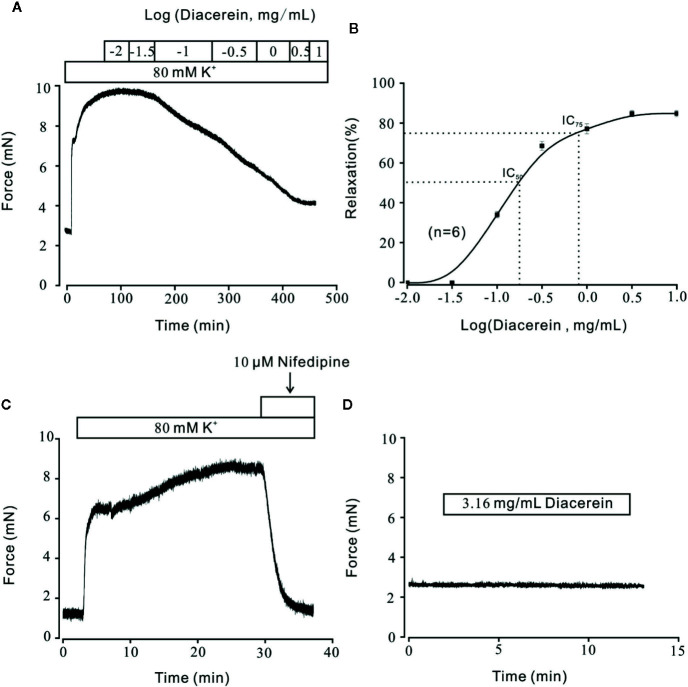
Relaxant effects of diacerein on high K^+^-precontracted mouse tracheal rings (mTRs). **(A)** A representative tension record indicated that high K^+^ induced a steady contraction which could be inhibited by 0.1~3.16 mg/ml diacerein in a dose-dependent manner. **(B)** Dose-relaxation curve of diacerein on high K^+^-precontracted mTRs (n = 6/6 mice). **(C)** High K^+^-induced precontraction was completely blocked by voltage-dependent L-type Ca^2+^ channel (VDLCC) specific blocker, 10 μM nifedipine (n = 6/6 mice). **(D)** 3.16 mg/ml diacerein had no effect on basal tone of mTRs (n = 6/6 mice).

Airway smooth muscle contractility is one of the unique symptoms of asthma (Hocking, 2002; Aghasafari et al., 2019; Sangaphunchai et al., 2020). The relaxant characteristic of rhein, an active metabolite of diacerein has been identified on rat intestinal muscle (Odenthal and Ziegler, 1988). To explore the potential relaxant characteristic of diacerein on mouse airway smooth muscle, a typical agonist, K^+^, which could evoke smooth muscle contraction gradually (Tan et al., 2017; Chen et al., 2019; Wen et al., 2020) was employed to precontract mTRs. In this experiment, 80 mM K^+^ was applied to effectively precontract mTRs (**Figure 1A**). It turned out that diacerein (0.1~3.16 mg/ml) could gradually inhibit high K^+^-induced precontraction on mTRs in a dose-dependent manner. According to the dose-contraction curve exhibited in **Figure 1B**, the half-maximal inhibitory concentration (IC_50_) and IC_75_ were 0.18 ± 0.067 and 0.86 ± 0.06 mg/ml, respectively. And the maximal relaxation was calculated as 81.02 ± 1.95%, while diacerein reached a maximum concentration (3.16 mg/ml). As well known, high K^+^ could evoke VDLCCs *via* depolarizing cell membrane (Yaguchi and Nishizaki, 2010). To confirm the participation of VDLCCs in relaxant activity of diacerein on precontracted mTRs induced by high K^+^, a selective blocker of VDLCCs, 10 μM nifedipine (Darnell and Peters, 1982) was employed and a similar inhibitory activity on high K^+^‐induced steady state contraction was also observed in mTRs (**Figure 1C**), which implied that blockade of VDLCCs might be involved in diacerein-induced relaxation on mTRs. These results indicated that diacerein inhibited high K^+^-induced pre-contraction in a dose-dependent way and VDLCCs might participate in the process. It should be noted that 3.16 mg/ml diacerein had no effect on resting mouse tracheal ring (**Figure 1D**).

### Diacerein Blocked High K^+^-Evoked Extracellular Ca^2+^ Influx *via* VDLCCs

**Figure 2:**
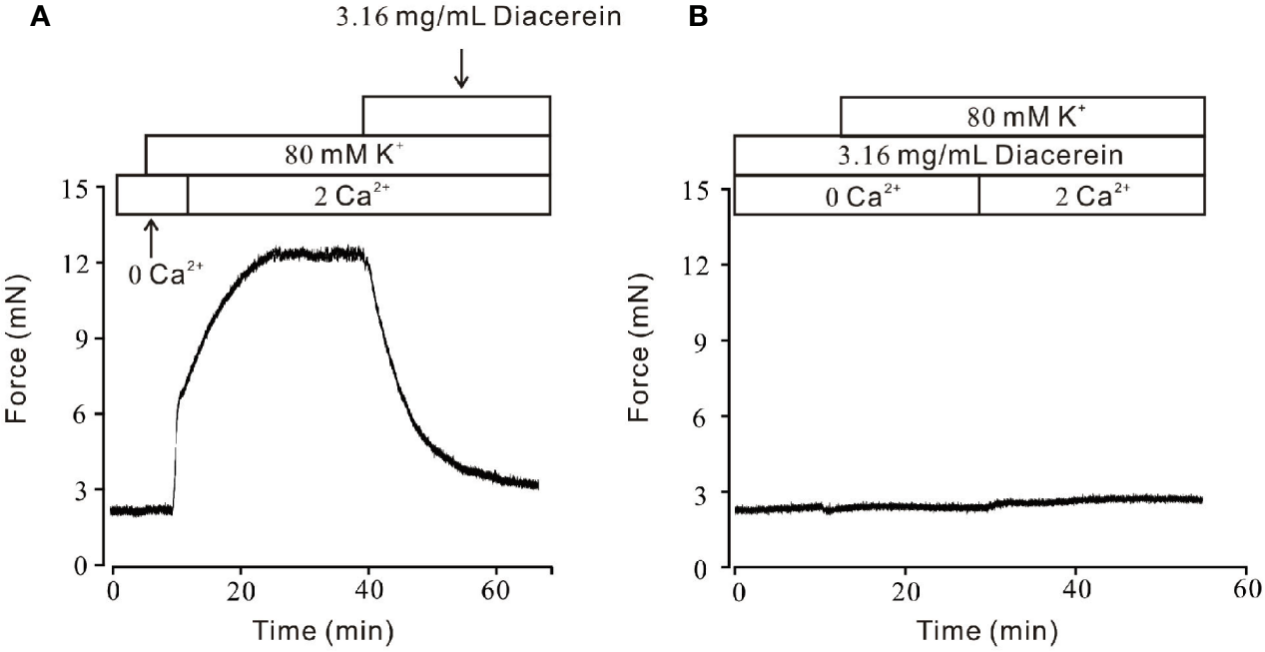
Diacerein blocked high K^+^-induced Ca^2+^ influx. **(A)** High K^+^ evoked a sustained contraction when the calcium concentration in solution switched from 0 to 2 mM. Subsequently, the contractile effect was almost completely erased by 3.16 mg/ml diacerein (n = 6/6 mice). **(B)** In the presence of 3.16 mg/ml diacerein, high K^+^ failed to induce contraction on mouse tracheal rings (TRs) either under Ca^2+^-free condition or after 2 mM Ca^2+^ restoration (n = 6/6 mice).

It has been well known that VDLCCs could mediate extracellular Ca^2+^ entry. To further clarify smooth muscle relaxant activity of diacerein shown in **Figure 1**, the following experiments were designed to explore whether calcium was involved in diacerein-induced relaxation on high K^+^-evoked precontracted mTRs. As shown in **Figure 2A**, In the 0 Ca^2+^ solution, high K^+^ couldn’t evoke contraction, indicating that extracellular calcium influx was indispensable for high K^+^-induced contraction. When Ca^2+^ was restored, high K^+^ subsequently evoked a steady contraction on mTRs, which was almost completely inhibited by 3.16 mg/ml diacerein. Furthermore, high K^+^ failed to induce contractions on mTRs in the presence of 3.16 mg/ml diacerein under 0 Ca^2+^ condition or subsequent 2 Ca^2+^ restoration (*p* > 0.05) (**Figure 2B**). The possible explanation was that the presence of diacerein has blocked VDLCCs, then high K^+^ could not evoke extracellular Ca^2+^ entry. These results further confirmed that Ca^2+^ mobilization was essential for diacerein-evoked relaxation of high K^+^-induced precontracted mTRs.

### Diacerein Blocked Voltage-Dependent L-Type Ca^2+^ Channels Currents

**Figure 3:**
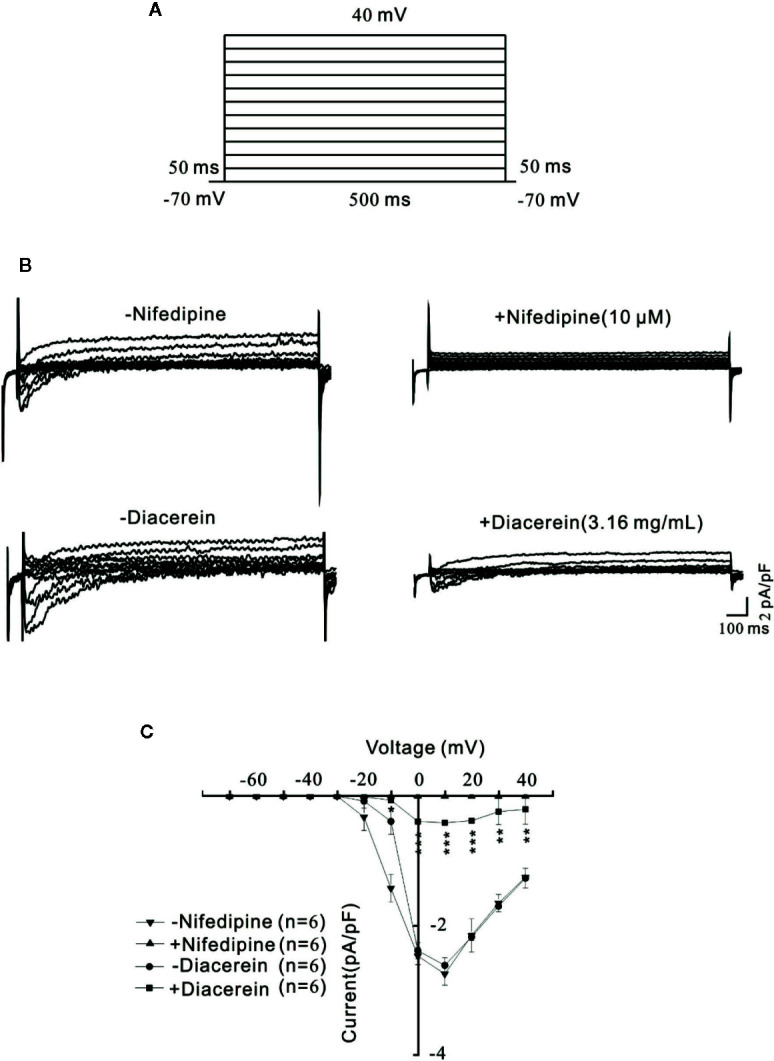
Diacerein blocked voltage-dependent L-type Ca^2+^ channel (VDLCC) currents on single mouse airway smooth muscle cell (mASMC). **(A)** The protocol used to measure VDLCC-mediated currents. **(B)** VDLCC currents could be abolished by 10 μM nifedipine or 3.16 mg/ml diacerein. **(C)** Current-voltage curve was constructed based on the results of six cells from six mice. NS, no significant, *, p < 0.05, **, p < 0.01, ***, p < 0.001.

To further explore the involvement of VDLCCs in the smooth muscle relaxant activity of diacerein, VDLCC currents were measured on single mASMC. As shown in **Figure 3A**, VDLCC currents were measured from −70 to +40 mV in 10 mV increments every 50 ms. A particular VDLCCs blocker, nifedipine was employed to eliminate the currents, which confirmed the record of VDLCC currents (**Figure 3B**, top). The same currents were also almost completely abolished by 3.16 mg/ml diacerein (**Figure 3B**, bottom). As indicated in the current-voltage (*I–V*) curve of VDLCC, diacerein could significantly erase the current amplitude of VDLCCs, which suggested that diacerein could inhibit VDLCC currents (**Figure 3C**). Taken together, these above results demonstrated that diacerein could relax high K^+^-induced smooth muscle contraction on mice by blocking the VDLCCs then suppress intracellular Ca^2+^.

### Diacerein Relaxed ACh-Induced Precontraction in a Dose-Dependent Manner

**Figure 4:**
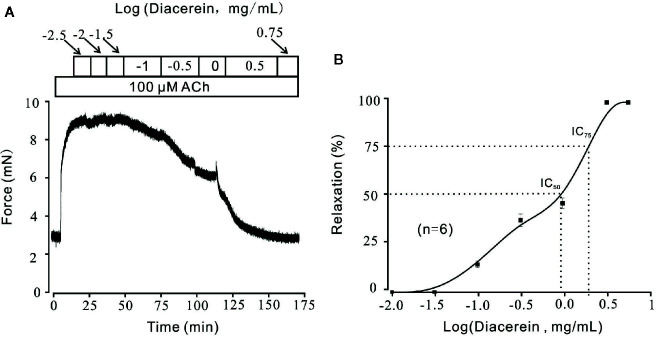
Relaxant effects of diacerein on ACh-precontracted mouse tracheal rings (mTRs). **(A)** A representative tension record indicated that ACh induced a steady contraction which could be inhibited by 0.1~3.16 mg/ml diacerein in a dose-dependent manner. **(B)** Dose-relaxation curve of diacerein on high K^+^-precontracted mTRs (n = 6/6 mice).

**Figure 5:**
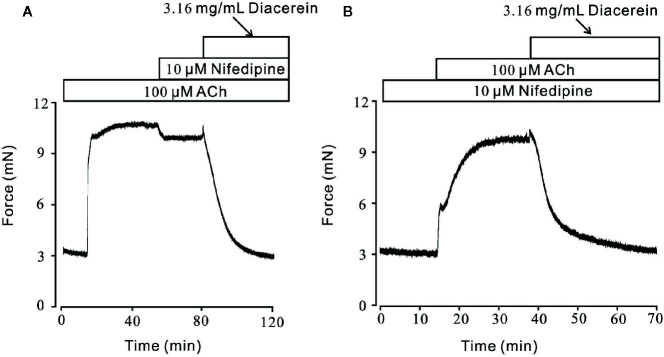
Diacerein relaxed ACh-induced precontraction on mouse tracheal rings (mTRs) after blocking voltage-dependent L-type Ca^2+^ channels (VDLCCs) with nifedipine. **(A)** ACh-induced contraction was partly reversed by 10 μM nifedipine. The remaining contraction was completely inhibited by 3.16 mg/ml diacerein, subsequently (n = 6/6 mice). **(B)** In the presence of 10 μM nifedipine, ACh-induced contraction was inhibited by 3.16 mg/ml diacerein (n = 6/6 mice).

Smooth muscle relaxation was a complicate electrophysiological process and various ion channels were involved and collaborated (Joseph et al., 2013). We sought to identify other ion channels besides VDLCC which might also participate in diacerein-induced relaxation. ACh, a well-known agonist, which was widely used in evoking contraction of smooth muscle through NSCCs and VDLCCs (Inoue and Isenberg, 1990; Dai et al., 2006) was also employed to precontract mTRs in current study. It turned out that 0.1-3.16 mg/ml diacerein could completely reverse 100 μM ACh-induced precontraction in a dose-dependent manner (**Figure 4A**). According to the concentration-relaxation curve shown in **Figure 4B**, the IC_50_ and IC_75_ were 0.75 ± 0.035 and 1.773 ± 0.058 mg/ml, respectively. The maximal relaxation was 98.00 ± 2.185%. To further identify the participation of NSCCs, VDLCCs were excluded with the specific antagonist nifedipine (**Figure 5**). As shown in **Figure 5A**, 100 μM ACh evoked a precontraction on mTR, which could be partially reversed by 10 µM nifedipine. Subsequently, additional 3.16 mg/ml diacerein could completely relax the sustained tension. Furthermore, 100 μM ACh-induced precontraction was also completely relaxed by 3.16 mg/ml diacerein in the presence of nifedipine (**Figure 5B**). These results indicated that besides VDLCCs, NSCCs were also participated in diacerein-induced relaxation. Also, it was found that the specific blocker of VDLCCs, 10 µM nifedipine-induced relaxation was much smaller than subsequent diacerein-induced relaxation (**Figure 5A**), which indicated that NSCCs might participate a more important role than VDLCCs.

To exclude the possible side effects of diacerein on airway smooth muscles, the reversibility and repeatability of relaxant characteristic exhibited by diacerein were studied on agonists-precontracted mTRs. As indicated in **Figure S1**, after washing out, diacerein succeeded to evoke a similar relaxation in the presence of high K^+^ or ACh. The results confirmed diacerein-induced smooth muscle relaxation was reversible and repeatable.

### Diacerein Blocked ACh-Evoked Intracellular Ca^2+^ Release and Extracellular Ca^2+^ Influx *via* Voltage-Dependent L-Type Ca^2+^ Channels and Non-Selective Cation Channels

**Figure 6:**
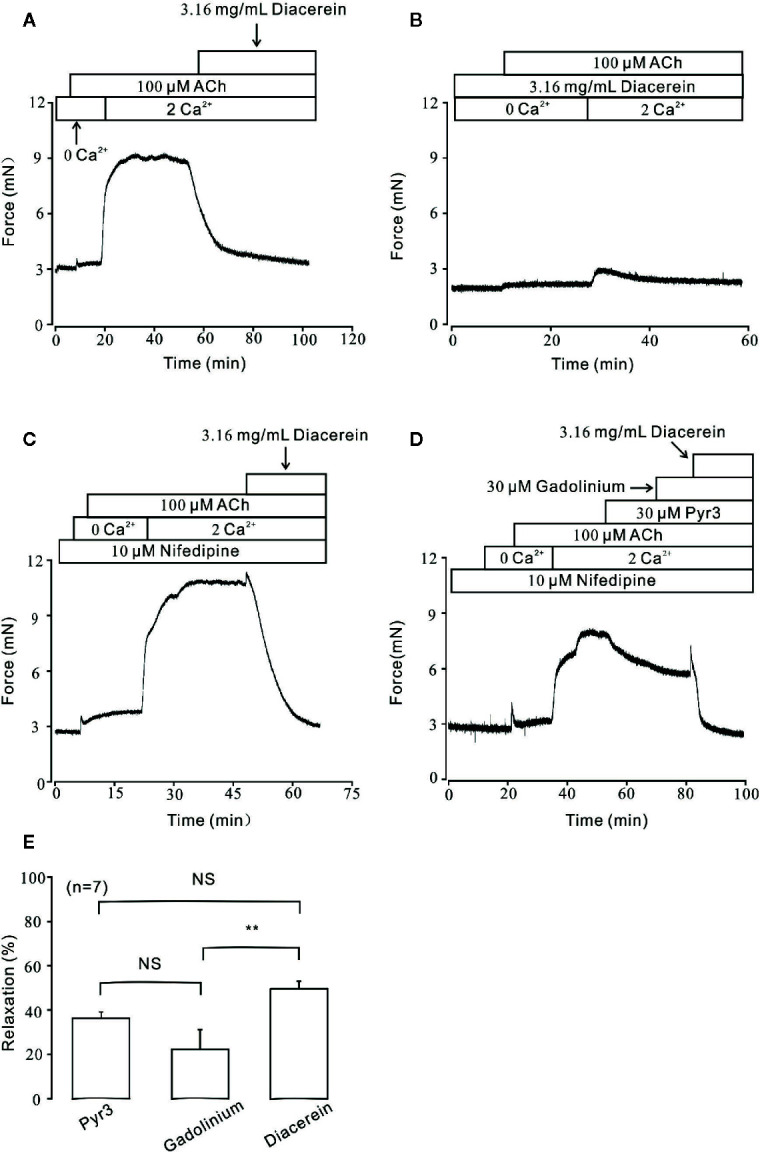
Diacerein blocked ACh-induced Ca^2+^ influx. **(A)** Under Ca^2+^-free condition, 100 μM ACh could evoke a small and transient contraction which indicated intracellular calcium was released. After the restoration of 2 mM Ca^2+^, a large and steady contraction was observed, and inhibited by 3.16 mg/ml diacerein subsequently. (n = 6/6 mice). **(B)** In the presence of 3.16 mg/ml diacerein, 100 μM ACh failed to induce contraction on mouse tracheal rings (TRs) either under Ca^2+^-free condition or after 2 mM Ca^2+^ restoration (n = 6/6 mice). **(C)** The similar 100 μM ACh-induced contraction and 3.16 mg/ml diacerein-induced relaxation as shown in (*A*) occurred when voltage-dependent L-type Ca^2+^ channels (VDLCCs) were excluded with 10 μM nifedipine (n = 6/6 mice). **(D)** In the presence of nifedipine, ACh-induced contraction in 2 Ca^2+^ solution could be reversed by 30 μM Pyr3, 30 μM gadolinium, and 3.16 mg/ml diacerein, sequentially (n = 7/7 mice). **(E)** The bar graph showed the average relaxant percentages of Pyr3, gadolinium, and diacerein, respectively. NS, no significant; **, p < 0.01.

Besides VDLCC, the increase of intracellular calcium during smooth muscle contraction was partly due to external calcium entry *via* NSCCs (Wynne et al., 2009). To further characterize the role of calcium in diacerein-induced relaxation, Ca^2+^ entry through NSCCs was studied under 100 μM ACh-induced pre-contraction. As shown in **Figure 6A**, under Ca^2+^-free condition, ACh induced a small and transient contraction, indicating that ACh could release internal stored Ca^2+^. Subsequently, the addition of 2 mM Ca^2+^ evoked a large and steady contraction in the presence of ACh, which was completely abolished by 3.16 mg/ml diacerein. However, in the presence of 3.16 mg/ml diacerein, ACh failed to induce transient or sustained contraction under 0 Ca^2+^ or 2 Ca^2+^ condition, respectively (**Figure 6B**). The possible explanation was that VDLCCs and NSCCs have been blocked by diacerein, leading to the failures of ACh-released intracellular calcium storage and ACh-evoked extracellular calcium entry. To isolate NSCCs, 10 μM nifedipine was employed to exclude VDLCCs in **Figures 6C, D**. In the presence of nifedipine, intracellular Ca^2+^ transiently released after the addition of 100 µM ACh in Ca^2+^-free medium. With the restoration of 2 mM Ca^2+^, a sustained contraction was induced and subsequently reversed by 3.16 mg/ml diacerein. It was well-known that elevated intracellular Ca^2+^ may result from an increased extracellular calcium influx through transient receptor potential channels (TRPCs), which were important components of NSCCs (Gonzalez-Cobos and Trebak, 2010). To further confirm the involvement of TRPCs in diacerein-induced relaxation, Pyr3 and gadolinium, two particular blockers of TRPCs (Glasnov et al., 2009; Mijares et al., 2014; Xiao et al., 2014) were used sequentially. As shown in **Figures 6D, E**, in the presence of nifedipine, the sustained contraction induced by ACh under 2 Ca^2+^ condition was abolished by 30 µM Pyr3, 30 µM gadolinium, and 3.16 mg/ml diacerein, respectively. The average relaxant percentages were 37.36 ± 5.34%, 20.09 ± 3.56%, and 52.4 ± 3.1%, respectively. These results indicated that diacerein could relax ACh-induced contraction through blockade of NSCCs and VDLCCs, then leading to intracellular Ca^2+^ decreases. Particularly, TRPCs might account for approximately 50 percent of the diacerein-induced calcium mobilization *via* NSCCs.

### Diacerein Blocked Non-Selective Cation Channels Currents

**Figure 7:**
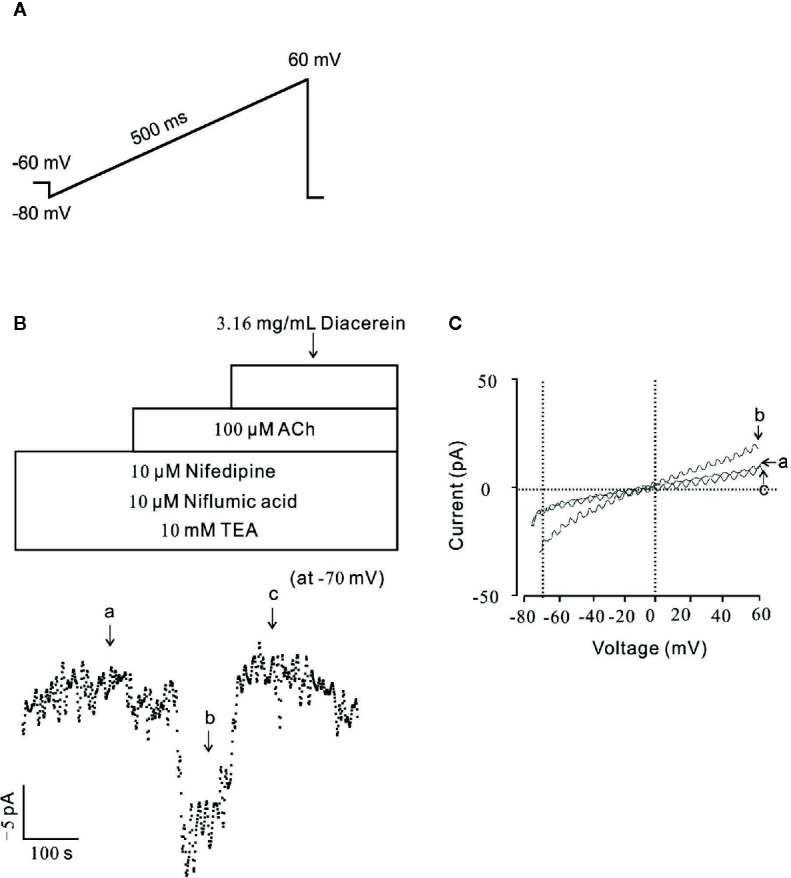
Diacerein blocked non-selective cation channels (NSCC) currents. **(A)** The ramp clamp protocol was employed to record NSCC currents in single airway smooth muscle cells (ASMC). **(B)** To isolate NSCC currents, VDLCC currents, Cl^−^ currents, and K^+^ currents were inhibited by 10 μM nifedipine, 10 μM NA, and 10 mM TEA, respectively. Then 100 μM ACh-induced NSCC currents was inhibited by 3.16 mg/ml diacerein (n = 6/6 mice). The values at −70 mV were obtained to plot current-time traces. **(C)** The net ramp currents at times a, b, and c.

To further identify the effect of diacerein on NSCC currents, whole-cell currents were recorded under a ramp voltage from −80 to +60 mV (**Figure 7A**). To isolate NSCC currents, currents from VDLCCs, Cl^−^ channels, and K^+^ channels were blocked by 10 µM nifedipine, 10 µM NA, and 10 mM TEA, respectively. As shown in **Figure 7B**, the isolated NSCC currents could be completely blocked by 3.16 mg/ml diacerein. Three representative ramp current traces at time points a, b, and c were shown in **Figure 7C**. This result indicated that diacerein could inhibit ACh-induced NSCC currents.

### Diacerein Switched Na^+^/Ca^2+^ Exchangers During Relaxation

**Figure 8:**
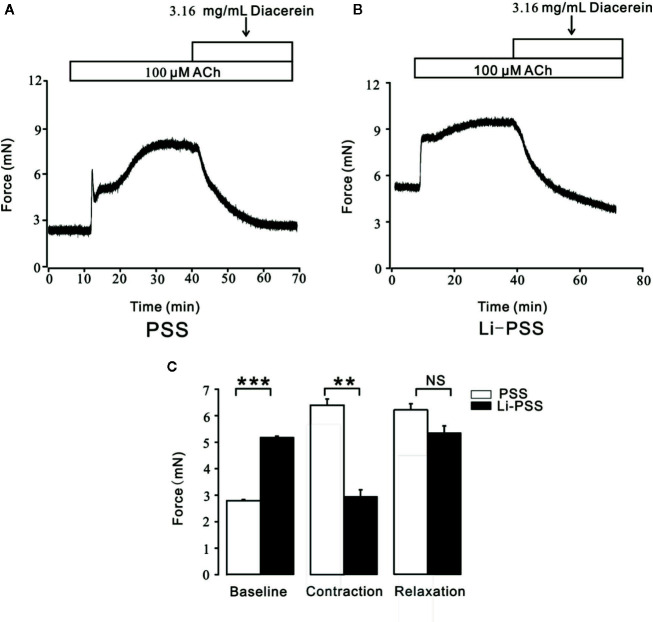
Diacerein switched Na^+^/Ca^2+^ exchangers (NCX). **(A)** 3.16 mg/ml diacerein attenuated 100 μM ACh-induced contraction under physiological salts solution (PSS) condition (n = 6/6 mice). **(B)** 3.16 mg/ml diacerein reversed ACh-induced contraction under Li-PSS condition. Statistically higher baseline, statistically smaller net contractile force, and non-significantly relaxant value were observed (n = 6/6 mice). **(C)** The bar graph showed the comparisons of net forces at the baseline, contraction, relaxation (n = 6/6 mice). NS, no significant; **, p < 0.01; ***, p < 0.001.

In addition to conventional Ca^2+^ permeable channels, NCX was a bi-directional membrane ion transporter which could be a pivotal way for Ca^2+^ entry into smooth cells (Dai et al., 2006; Khananshvili, 2014). To explore the potential role of NCX during diacerein-evoked relaxation, PSS and Li-PSS without sodium were parallelly and separately applied. As shown in **Figure 8B**, under the condition of Li-PSS, ACh-induced a steady contraction with a significantly higher basal tone compared with that under PSS condition (**Figure 8A**), which indicated that without extracellular sodium, NCX reversed and transported calcium into smooth muscle cell to increase intracellular Ca^2+^. The process was known as “Ca^2+^ influx/Na^+^ outflow” mode, or “reverse” mode. As a result, the net contractile force induced by ACh-evoked Ca^2+^ influx in Li-PSS was statistically smaller than that in PSS. Then 3.16 mg/ml diacerein was added. It turned out that diacerein-elicited relaxation showed no significant difference in PSS or Li-PSS, which implied that in the presence of diacerein, pre-reversed NCX could be switched to “calcium exit” mode, also known as “forward” mode to decrease intracellular calcium ion. The bar graph was showed at **Figure 8C**. This result indicated the potential operated mechanism of NCX during diacerein-induced relaxation.

### Diacerein Activated K^+^ Channels, Especially Large-Conductance Ca^2+^-Activated K^+^ Channels

**Figure 9:**
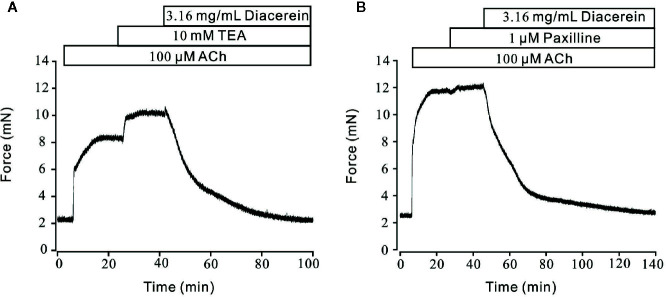
Diacerein activated K^+^ channels. **(A)** 100 μM ACh-evoked contraction was significantly enhanced by 10 mM tetraethylammonium chloride (TEA). Addition of 3.16 mg/ml diacerein completely relaxed the contractile mouse tracheal rings (mTRs) (n = 6/6 mice). **(B)** 100 μM ACh-evoked contraction was significantly enhanced by 1 μM paxilline. Addition of 3.16 mg/ml diacerein completely relaxed the contractile mTRs (n = 6/6 mice).

Unlike the well-defined contractile roles of calcium channels, activated potassium channels might also play an important part in regulation of smooth muscle tone by hyperpolarizing cell membrane, then favoring relaxation (Ko et al., 2008; Jackson, 2018). Therefore, the role of K^+^ channel in diacerein-induced relaxation was tested (**Figure 9**). As shown in **Figure 9A**, 100 μM ACh evoked-contraction could be significantly enhanced by 10 mM TEA, an inhibitor of K^+^ channels, which indicated that blockade of K^+^ channel could strengthen contraction. Subsequently, the total contraction was completely abolished by 3.16 mg/ml diacerein, which indicated that blocked K^+^ channel was activated during diacerein-induced relaxation. Several studies have revealed that BK channel, a classical type of potassium channels, constituted a key physiological feature of regulating smooth muscle tone by affecting cellular Ca^2+^ mobilization (Rothberg, 2012). To identify the role of BK channel in diacerein-induced relaxation, paxilline, a specific inhibitor of BK channels was applied in **Figure 9B**. It turned out that 100 μM ACh-induced contraction was enhanced by 1 μM paxilline. Then 3.16 mg/ml diacerein was added. Resultantly, the contraction was completely relaxed, which implied that the blocked BK channel was reactivated. These results demonstrate that K^+^ channels, especially BK channel, were involved in diacerein-induced relaxation.

### Diacerein Enhanced Large-Conductance Ca^2+^-Activated K^+^ Currents

**Figure 10:**
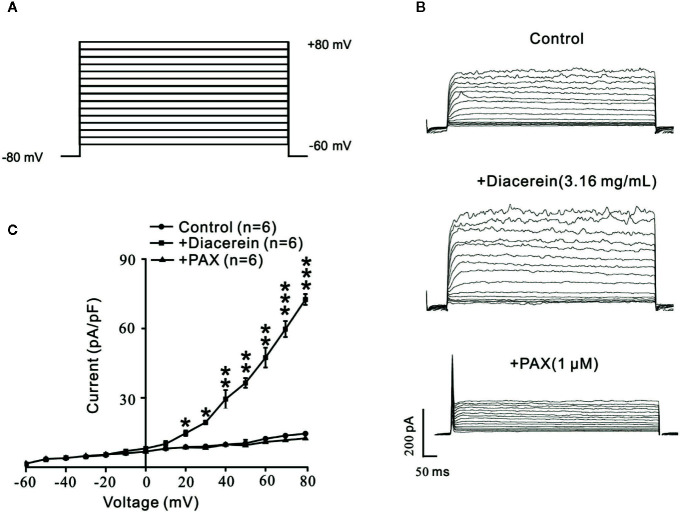
Diacerein strengthened K^+^ currents. **(A)** Large-conductance Ca^2+^-activated K^+^ (BK) currents was recorded under the ramp voltage ranged from −80 to +80 mV at 10 mV increments. **(B)** Representative recording of K^+^ currents under the conditions of control (upper), 3.16 mg/ml diacerein (middle), and 1 μM paxilline (lower) at different voltages. **(C)** Current-voltage curve was constructed based on the results of six cells from six mice. *, p < 0.05; **, p < 0.01; ***, p < 0.001.

To further identify the influence of diacerein on BK currents, the certain currents were measured with whole-cell recording method under the voltage ranged from −80 mV to +80 mV (**Figure 10A**). It turned out that BK currents were successfully recorded (**Figure 10B**, upper). The amplitudes of BK currents were significantly increased by 3.16 mg/ml diacerein (**Figure 10B**, middle), while the amplitudes were completely inhibited by 1 μM paxilline (**Figure 10B**, bottom). The bar graph was shown in **Figure 10C**. These results demonstrated that diacerein could strengthen BK currents in airway smooth muscle.

### Diacerein Reduced Respiratory System Resistance

**Figure 11:**
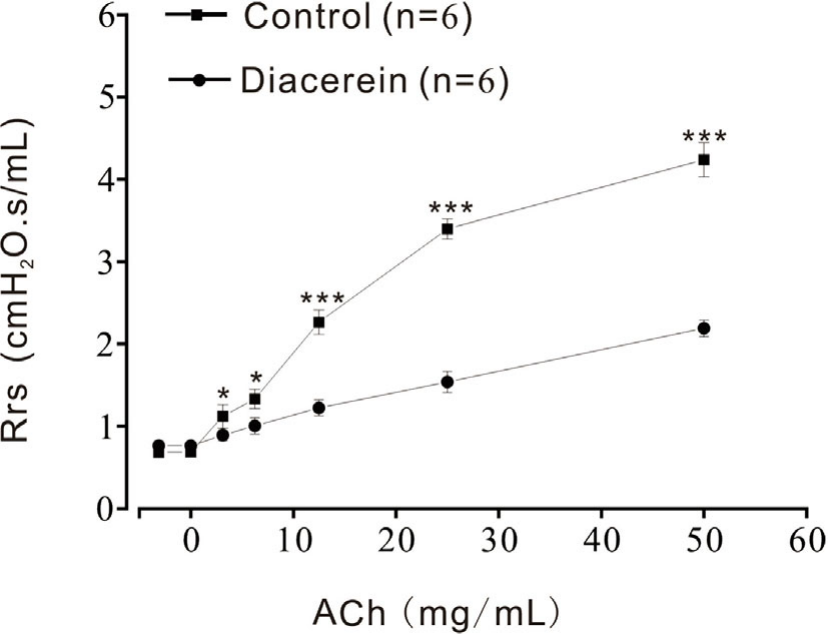
Diacerein inhibited respiratory system resistance (Rrs) increases. ACh-induced increases of Rrs in mice were significantly inhibited by diacerein (n = 6/6 mice). *, p < 0.05; ***, p < 0.001.

Above results identified that diacerein could relax precontracted mTRs. To further investigate the relaxant property of diacerein on mouse airway *in vivo*, Rrs was detected by the forced oscillation technique (**Figure 11**) with aerosolized ACh, which was added at a gradually increased concentration (3.125, 6.25, 12.5, 25, and 50 mg/ml). It turned out that Rrs was gradually increased by ACh in a dose-dependent manner, which could be significantly inhibited by 31.6 mg/ml diacerein. This result confirmed that diacerein could inhibit mouse airway contraction *in vivo*.

### Diacerein Reduced Systematic Inflammation and Mucus Secretion *In Vivo*

**Figure 12:**
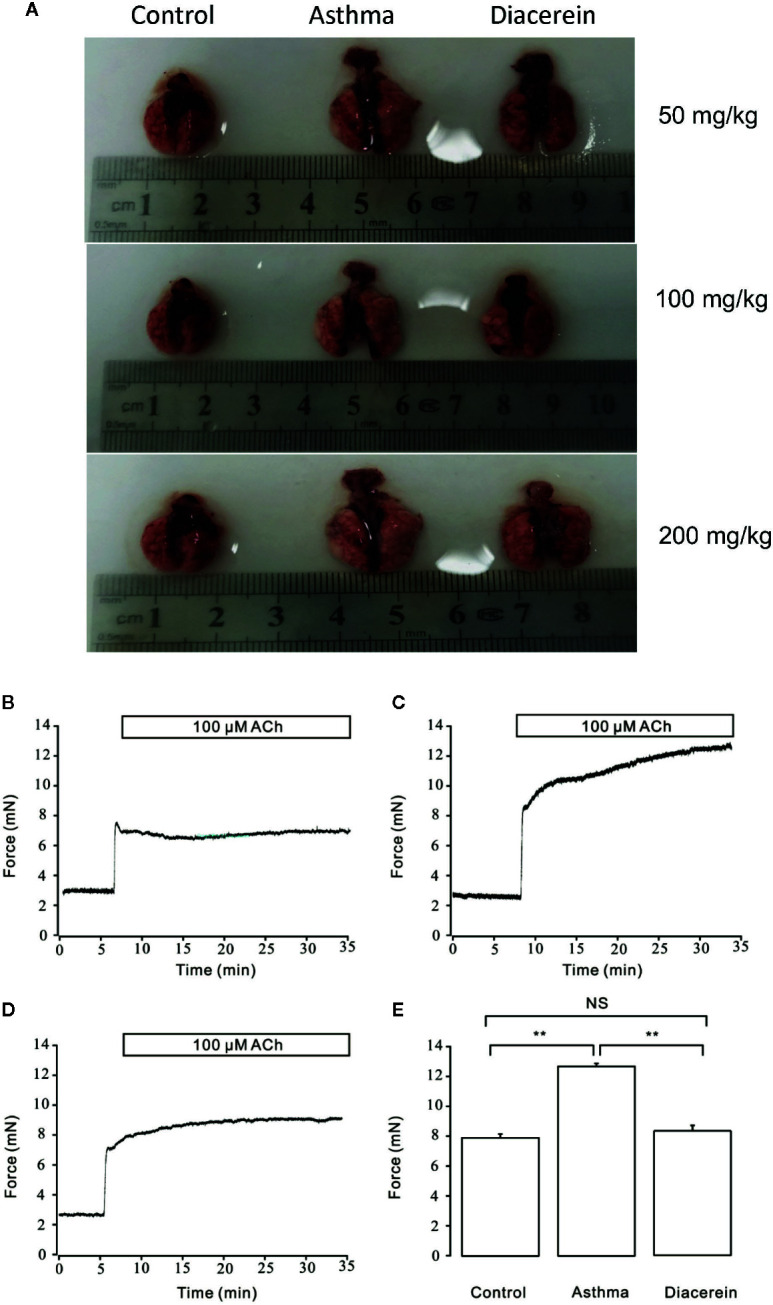
Asthmatic mice models were established with or without diacerein treatment. **(A)** Representative pictures of isolated lungs from control, asthma, and diacerein groups (50, 100, 200 mg/kg). **(B–D)** 100 μM ACh induced stable contractions on tracheas isolated from control, asthma, and diacerein groups, respectively. **(E)** The bar graph showed the comparison of contractile forces among control, asthma, and diacerein groups (n = 6/6 mice). NS, no significant; **, p < 0.01.

To further investigate the potential asthmatic treatment property of diacerein, asthmatic mice models with or without diacerein treatment (50, 100, 200 mg/kg) were established. As shown in **Figure 12A**, trachea and lung isolated from asthmatic mice exhibited typical pathological changes such as visible enlargement, swelling, and inflammation compared with control and diacerein groups. Then 50 mg/kg diacerein group was selected for further studies. Airway hyperresponsiveness, a typical symptom of asthma was further detected *via* tension measurement. 100 μM ACh was employed to trigger airway contractions in control, asthma, and diacerein groups, respectively (**Figures 12B–D**). It turned out that ACh-induced contraction on tracheal rings isolated from asthmatic mice was significantly larger compared with control and asthma groups. Meanwhile, there was no statistical difference between control group and diacerein group (**Figure 12E**). These results primarily indicated asthma model was successfully established. Furthermore, diacerein treatment could effectively reduce airway hypertension.

**Figure 13:**
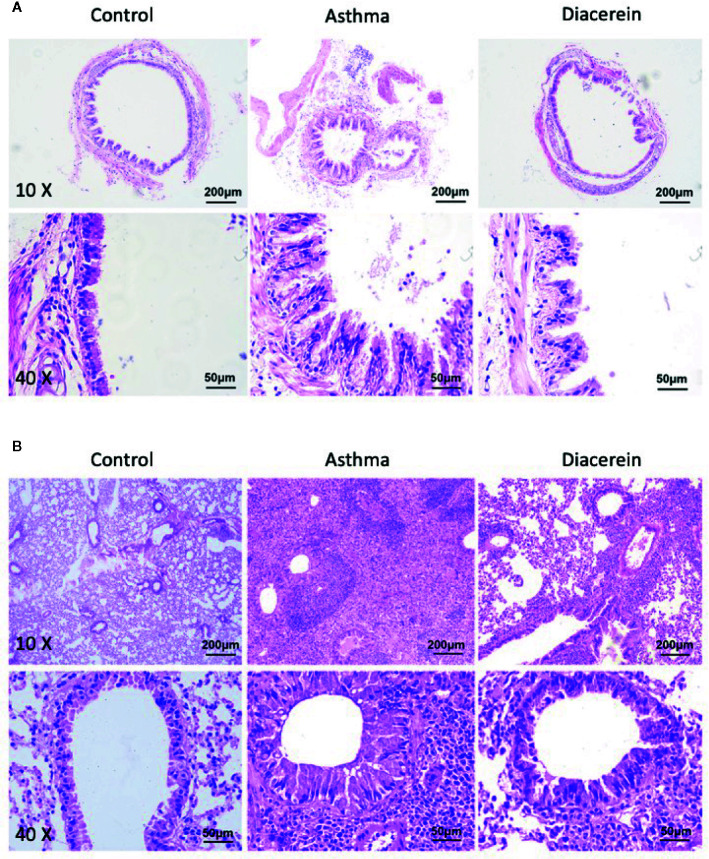
H&E staining showed that diacerein reduced airway inflammation. **(A)** Representative tracheal images were obtained from control, asthma, and diacerein group. **(B)** Representative lung images were obtained from control, asthma, and diacerein groups.

Next, the effect of diacerein on OVA-challenged asthma was studied on trachea and lung specimens isolated from control, asthma, diacerein groups with H&E staining and PAS staining. It turned out that in asthma group, the trachea was significantly narrowed and the structural integrity of ciliated epithelium were severely disturbed, while bronchial thickening and inflammation were both observed in lung sections (**Figure 13**, middle panel). To the opposite, diacerein group exhibited anti-inflammatory property to significantly reduce inflammation and repair damaged airway (**Figure 13**, right panel). Meanwhile, the control mice did not develop any airway inflammation (**Figure 13**, left panel). These results indicated that diacerein treatment could improve airway remodeling in asthmatic mice.

**Figure 14:**
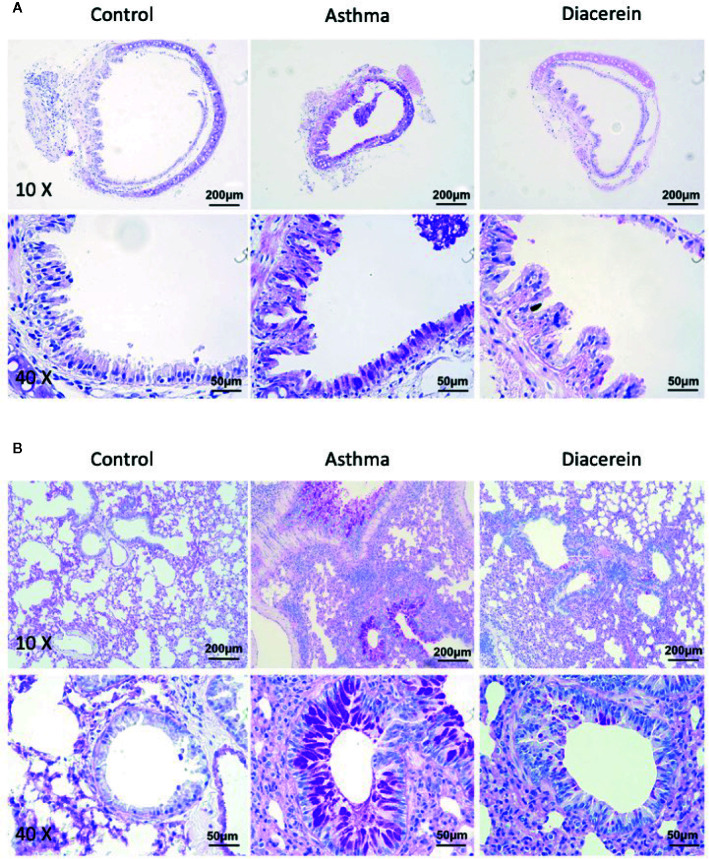
Periodic acid-Schiff (PAS) staining showed that diacerein reduced airway mucus hypersecretion. **(A)** Representative tracheal images were obtained from control, asthma, and diacerein group. **(B)** Representative lung images were obtained from control, asthma, and diacerein groups.

Then PAS staining was applied to identify stored mucins in airway goblet cells. It turned out that in asthma group, the tracheal epithelium exhibited severe mucous metaplasia, while PAS-stained mucins in goblet cells was significantly increased in lung specimens (**Figure 14**, middle panel). Meanwhile, diacerein group showed a significant reduction in PAS-labeled mucins, suggesting that diacerein could decrease mucus secretion in asthmatic group (**Figure 14**, right panel). The control group had no mucus secretion (**Figure 14**, left panel). All these results demonstrated that *in vivo* diacerein could relieve asthmatic symptoms such as airway remodeling, pulmonary inflammation and mucus secretion.

**Figure 15:**
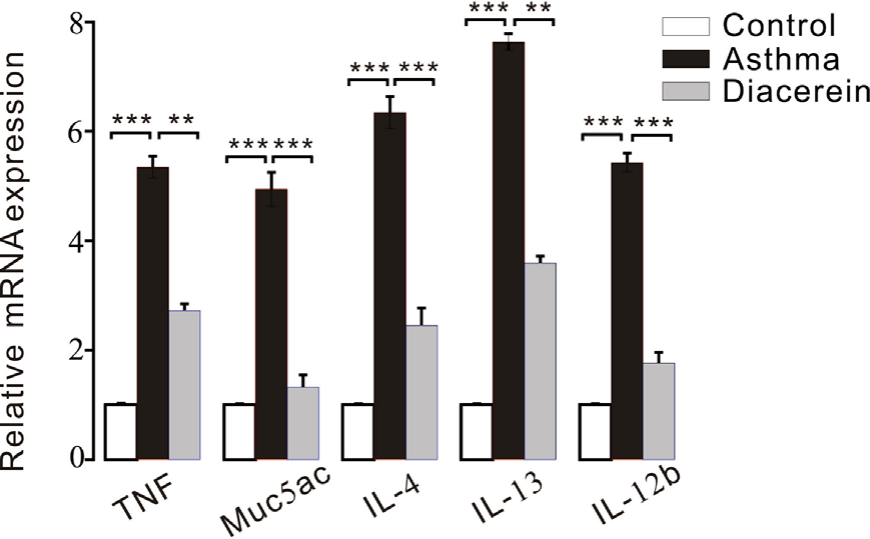
The bar graph showed the different expression levels of tumor necrosis factor (TNF), Muc5ac, interleukin (IL)-4, IL-13, IL-12b in control, asthma, and diacerein groups (n = 5/5 mice). **, p < 0.01; ***, p < 0.001.

Furthermore, RT-PCR was employed to detect the possible involved inflammatory factors. It turned out that the mRNA expressions of several inflammatory factors, such as tumor necrosis factor (TNF), Muc5ac, IL-4, IL-13, and IL-12b were significantly upregulated in asthma group compared with control and diacerein groups (**Figure 15**).

## Discussion

As one of the most common airway diseases, asthma affects almost 30 million people in China and more than 300 million people worldwide. Hundreds of drugs have been applied in asthma treatment (Bahadori et al., 2009; Moorman et al., 2012; Beasley et al., 2015; Becker and Abrams, 2017; Chen et al., 2018). However, new drugs are needed since those currently available could not effectively control symptoms and exacerbations in all patients and might cause severe side effects (McKeever et al., 2013; Kapadia et al., 2016; Brode et al., 2017; Fajt and Wenzel, 2017; Heffler et al., 2018). To accelerate the innovation of new therapies, there is a high prevalence of usage of already-approved drugs. Diacerein, a synthesized anthraquinone, has displayed anti-inflammatory property in OA treatment (Fidelix et al., 2014; Pavelka et al., 2016), while several studies have evaluated its anti-contractile effects on murine colon and uterus (Odenthal and Ziegler, 1988). These studies shed light on the possible therapeutic role of diacerein on typical symptoms of asthma, including abnormal ASM contractility and systemic inflammation.

In current study, the anti-contractile feature of diacerein was firstly explored on isolated mouse ASM. It was found that diacerein could significantly relax precontracted mouse ASM in a concentration-dependent way. Next, we sought to explore the underlying mechanism. Smooth muscle relaxation was a complicate electrophysiological process involving various ion channels which were expressed within ASM cells regulating the membrane potential, intracellular Ca^2+^ concentration, and smooth muscle contractility. Tension measurement with the presence of specific antagonists of certain ion channels indicated that diacerein could block VDLCCs and NSCCs, switch NCX, and activate K^+^ channel to regulate calcium mobilization. Among all these ion channels, it was found that NSCCs especially TRPCs might play a major role than VDLCCs in diacerein-induced calcium mobilization. Patch clamp recording further confirmed that diacerein could reduce VDLCC and NSCC currents and enhance BK currents. Taken together, a series of *in vitro* experiments revealed that blockade of Ca^2+^ influx through switched VDLCCs, NSCCs, BK channel, and NCX was essential in diacerein-induced smooth muscle relaxation. The potential mechanism of diacerein-induced relaxation might be that activated BK channels hyperpolarized the membrane potential of smooth muscle, which inhibited calcium entry through blocking calcium permeable channels, such as VDLCCs and NSCCs, and thereby relaxed through elimination of intracellular calcium. Meanwhile, NCX also played a partial role in this process to transport calcium.

Next, anti-contractile and anti-inflammatory effects of diacerein were explored at the individual levels. Measurements of respiratory resistance and airway tension of mice models further confirmed that diacerein could relieve airway hypertension and bronchospasm on mice. To further explore the pathological changes of tracheas and lungs in the asthmatic mice with or without diacerein treatment, H&E and PAS staining were applied to detect tissue inflammation and mucus secretion, respectively. Histological analysis showed that airway narrowing, smooth muscle hyperplasia, systematic inflammation, and mucus secretion were significantly relieved in the asthma mice treated with diacerein. To identify possible molecular mechanism, mRNA expressions of a series of inflammatory mediators were detected. Among them, mast cell-derived TNF contributed significantly to the OVA-induced airway inflammation (Nakae et al., 2007). Muc5ac was an important integral component of airway mucus (Bonser and Erle, 2017). The cytokines (IL-4 and IL-13) and pro-inflammatory cytokine (IL-12b) promoted cellular inflammation in the asthmatic lung (Oh et al., 2010; Li et al., 2017). The RT-PCR results showed that the increased expression levels of TNF, Muc5ac, IL-4, IL-13, and IL-12b in asthmatic mice was significantly reduced in diacerein-treated mice, which elucidated that diacerein might provide anti-inflammatory benefit *via* reducing these inflammatory mediators. Besides that, previous study also demonstrated that inflammatory cytokines might enhance ASM contraction by increasing expressions of certain ion channels and elevating intracellular calcium concentrations (Ding et al., 2019), which implied that the anti-contractile feature of diacerein might also benefit from down-regulated inflammatory mediators. However, more experiments need to be conducted to confirm it.

Through current study, the anti-inflammatory characteristic of diacerein has been extended to relaxant effect on asthmatic mice and the underlying molecular mechanism has been primarily clarified. However, there is still some limitations. We only reached an initial conclusion that NSCCs especially TRPCs might play a more important role in diacerein-induced relaxation. The most important ion channel through which diacerein mediates calcium mobilization needs to be figured out in further study. Meanwhile, we also found that several inflammatory mediators were down-regulated in diacerein-treated asthmatic mice. And the underlying signal pathway through which the inhibited inflammation factors relieve asthma symptoms needs to be further explored. Furthermore, the clinical significance of diacerein on asthma treatment needs to be confirmed in clinical trial.

## Conclusions

In summary, our research indicated that hypertension of mouse airway smooth muscle could be relaxed by diacerein *via* calcium mobilization which was mediated by VDLCCs, NSCCs, BK channels, and NCX. Furthermore, diacerein could effectively relieve typical asthmatic symptoms including airway thickening, airway remodeling, systematic inflammation, and mucus secretion *via* reduction of inflammatory mediators. The combination of anti-contractile and anti-inflammatory effects suggests that diacerein may represent an important new advance in the treatment of asthma especially as a potential bronchodilator. However, further clinical investigation is necessary.

## Data Availability Statement

The raw data supporting the conclusions of this article will be made available by the authors, without undue reservation.

## Ethics Statement

The animal study was reviewed and approved by Animal Care and Ethics Committee of the South-Central University for Nationalities.

## Author Contributions

JS and Q-HL conceived and designed the experiments. SS, SH, HQ, YP, and PZ performed the experiments. JS, SS, and LX analyzed the data and generated the figures. JS and LX wrote the manuscript.

## Funding

This project was supported by the National Natural Science Foundation of China (Grant No. 31771274) to Jinhua Shen and the Fund for Key Laboratory Construction of Hubei Province (Grant No. 2018BFC360). It was also supported by the Natural Science Foundation of Hubei Province, China (Grant No. 2018CFB594 to LX) and the China Scholarship Council (No. 201808420069 to LX).

## Conflict of Interest

The authors declare that the research was conducted in the absence of any commercial or financial relationships that could be construed as a potential conflict of interest.

## Supplementary Material

Figure S1 Diacerein has no harmful effect on mTRs bioactivity. **(A)** High K^+^ induced a steady-state contraction in a mTR, which was partially inhibited by 3.16 mg/mL diacerein. After washing out, a similar contraction was evoked by high K^+^ (n = 6/6 mice). **(B)** The bar graph showed no significant differences between forces at the control and wash-out stage. **(C)** 100 μM ACh-induced contraction could be almost completely inhibited by 3.16 mg/mL diacerein. After washing out, a similar contraction was evoked by 100 μM ACh. **(D)** The bar graph showed no significant differences between initial contraction and contraction after wash-out. NS, no significant.

## Abbreviations

ACh, acetylcholine chloride; Al(OH)_3_, aluminum hydroxide; BK channel, large-conductance Ca^2+^-activated K^+^ channel; BSA, bovine serum albumin; CsCl, cesium chloride; DTT, dithiothreitol; H&E, hematoxylin and eosin; IC_50_, half-maximal inhibition; HPA, hypothalamic-pituitary-adrenal; IL-1, interleukin 1; IP, intraperitoneally; JAK-STAT, Janus kinase-signal transducers and activators of transcription; mASMCs, mouse airway smooth muscle cells; mTRs, mouse tracheal rings; NA, niflumic acid; NCX, Na^+^/Ca^2+^ exchangers; NSCCs, non-selective cation channels; NSS, normal saline solution; OA, osteoarthritis; OVA, ovalbumin; PAS, periodic acid-Schiff; PAX, paxilline; PEEP, positive end expiratory pressure; PSS, physiological salts solution; Pyr3, pyrazole 3; Rrs, respiratory system resistance; SD, standard deviation; SPF, specific pathogen-free; TEA, tetraethylammonium chloride; TRPCs, transient receptor potential channels; VDLCCs, voltage-dependent L-type Ca^2+^ channels.
