# Supplementary material for: Anti-Contractile and Anti-Inflammatory Effects of Diacerein on Isolated Mouse Airways Smooth Muscle and Mouse Asthma Model
Source: Front Pharmacol. 2020 Sep 4;11:560361. doi: 10.3389/fphar.2020.560361 (PMC7498646; doi:10.3389/fphar.2020.560361)

Figure 13A Asthma 10X

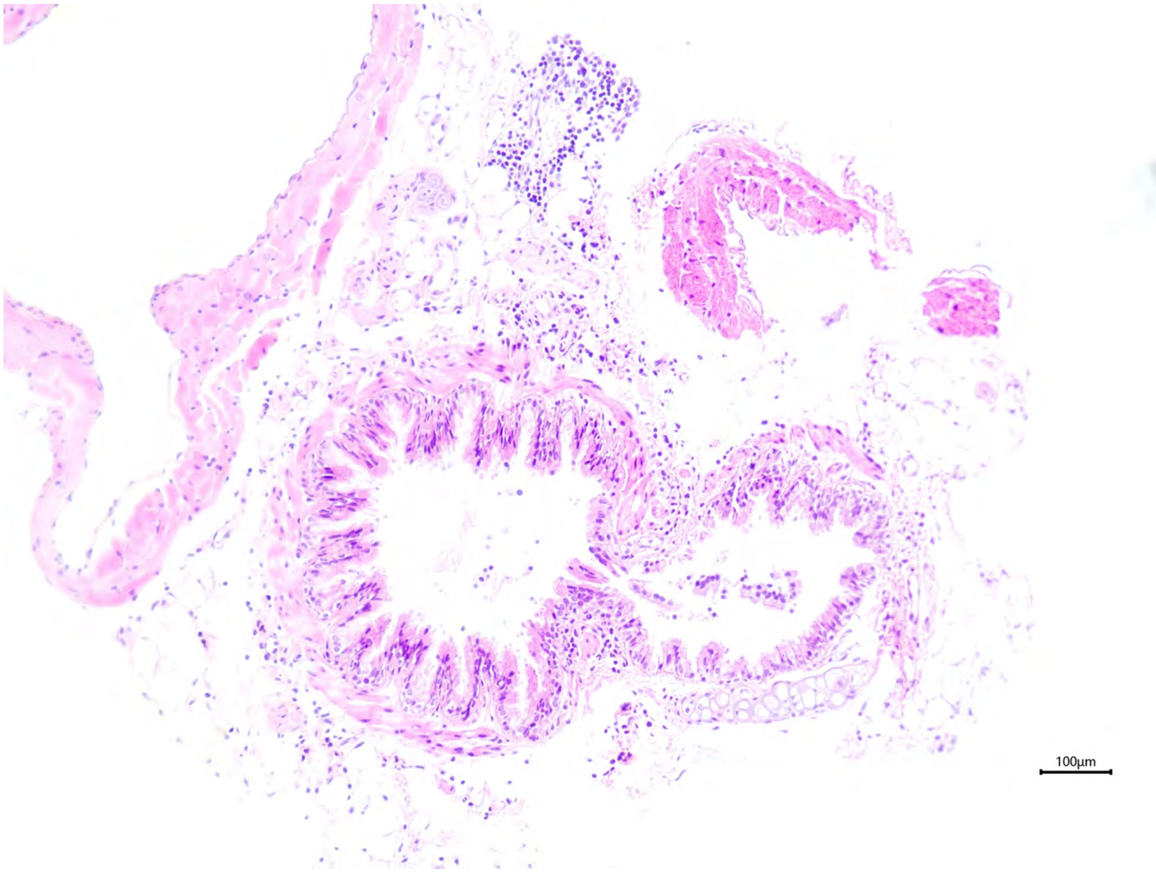

Figure 13A Asthma 40X

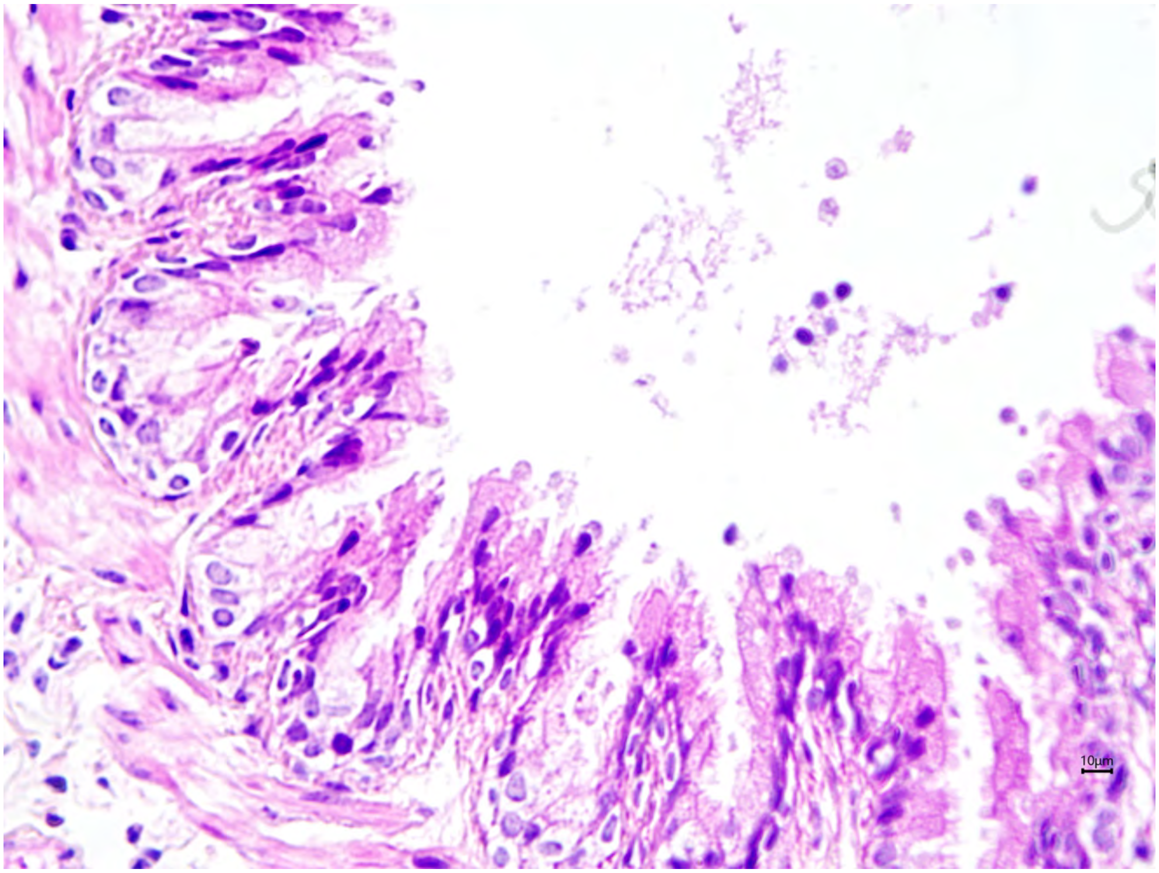

Figure 13A Control 10X

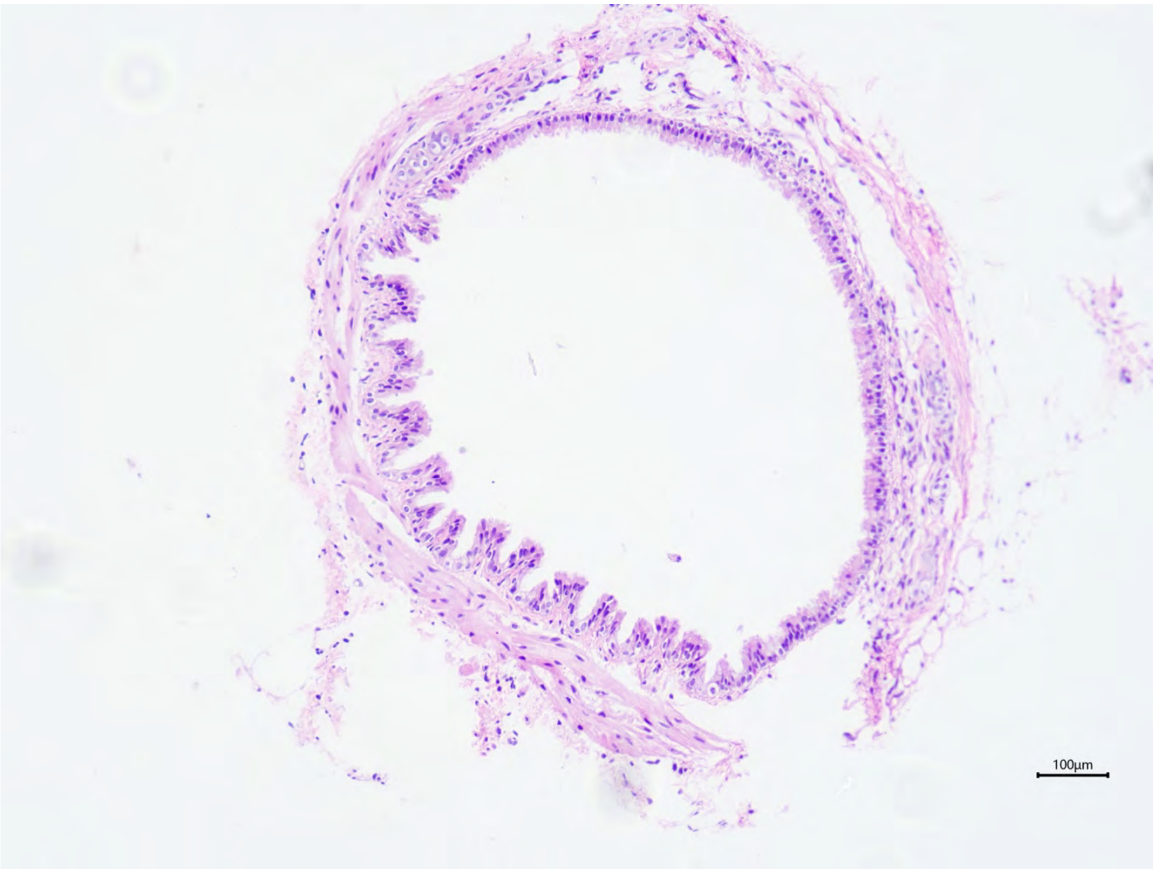

Figure 13A Control 40X

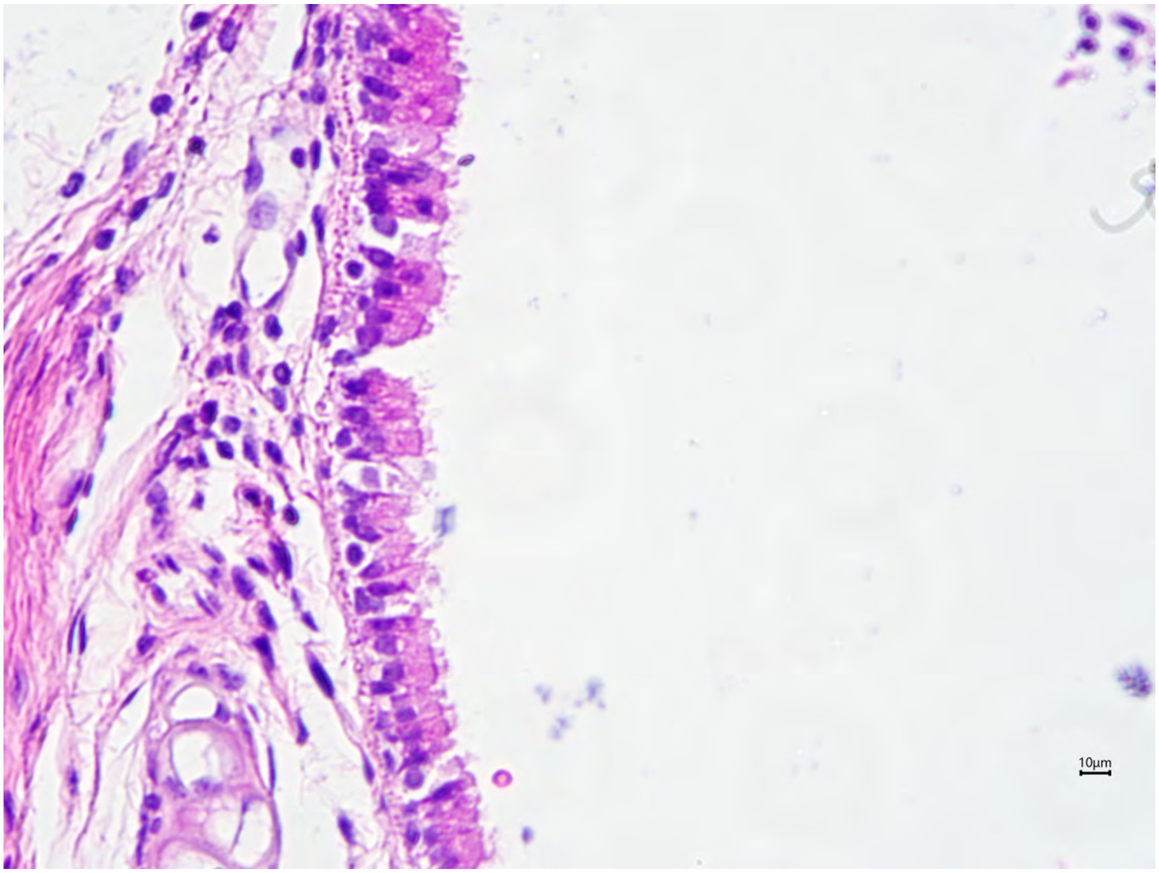

Figure 13A Diacerein 10X

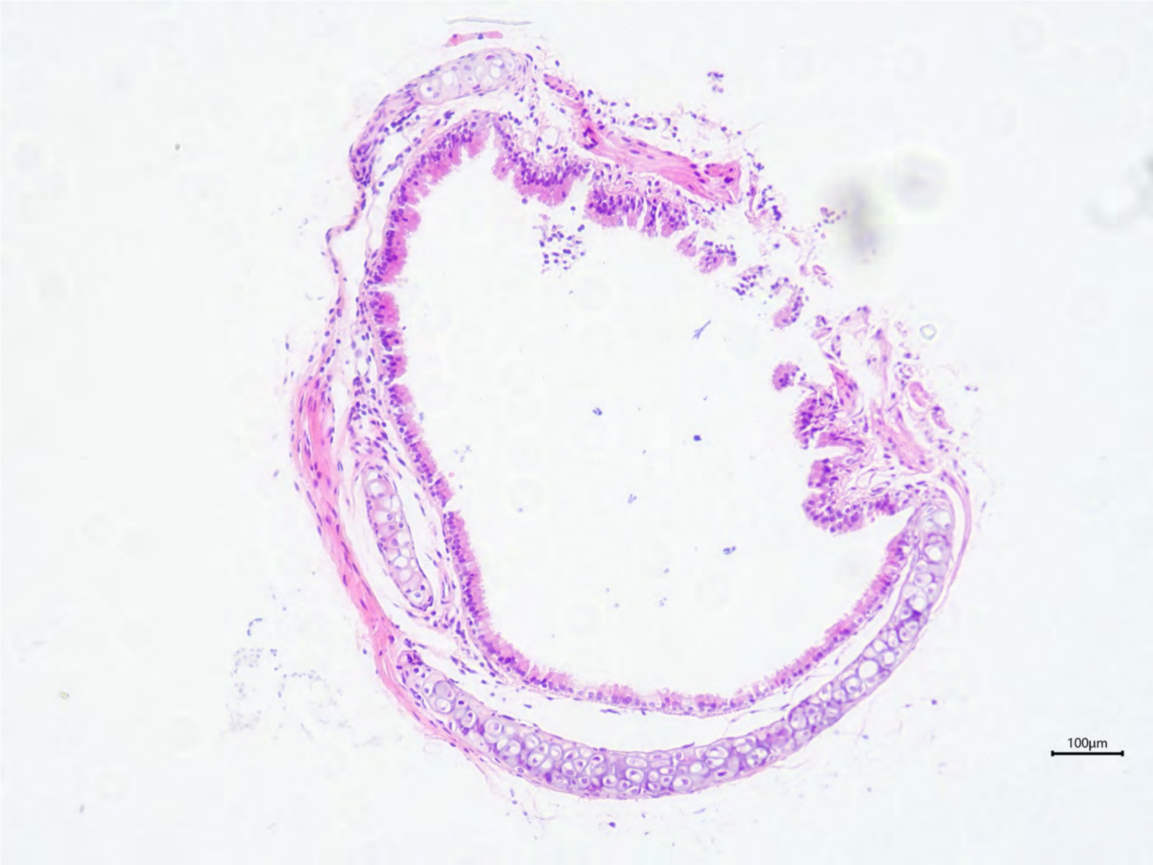

Figure 13A Diacerein 40X

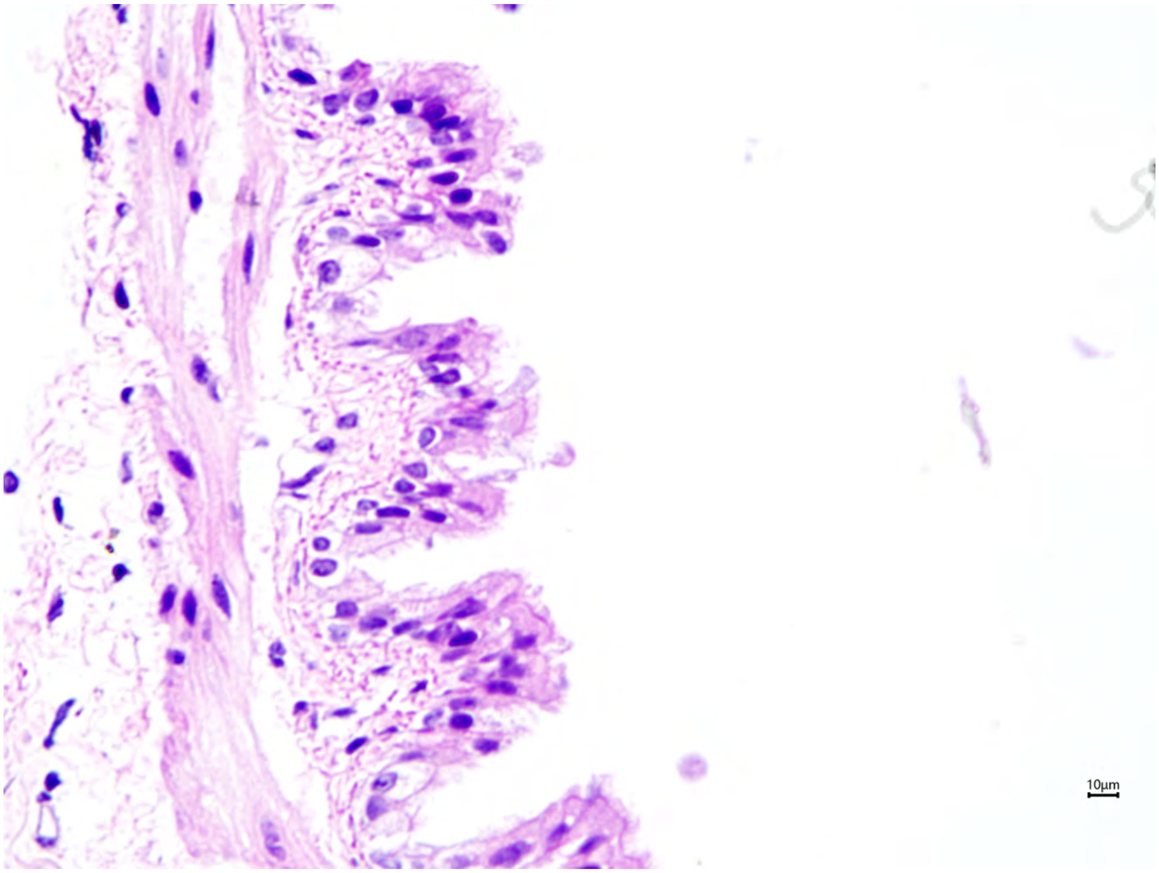

Figure 13B Asthma 10X

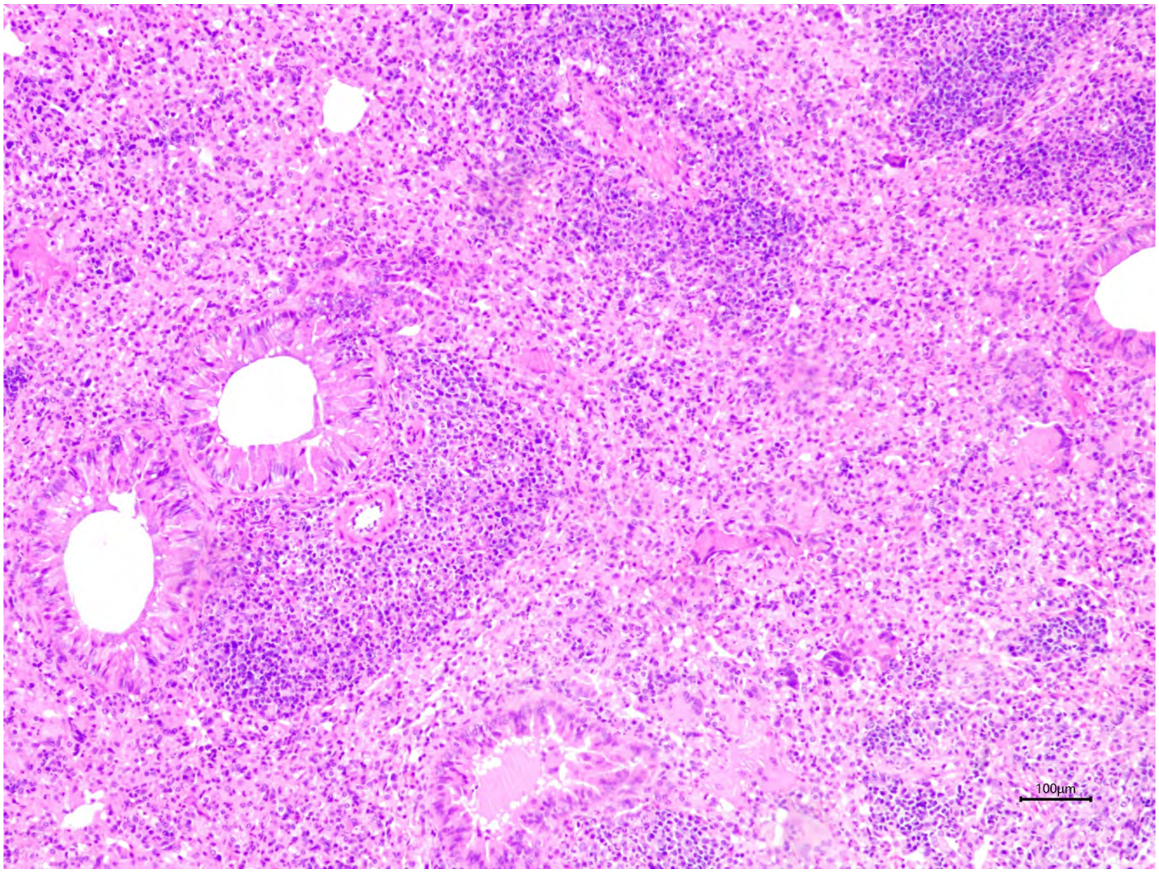

Figure 13B Asthma 40X

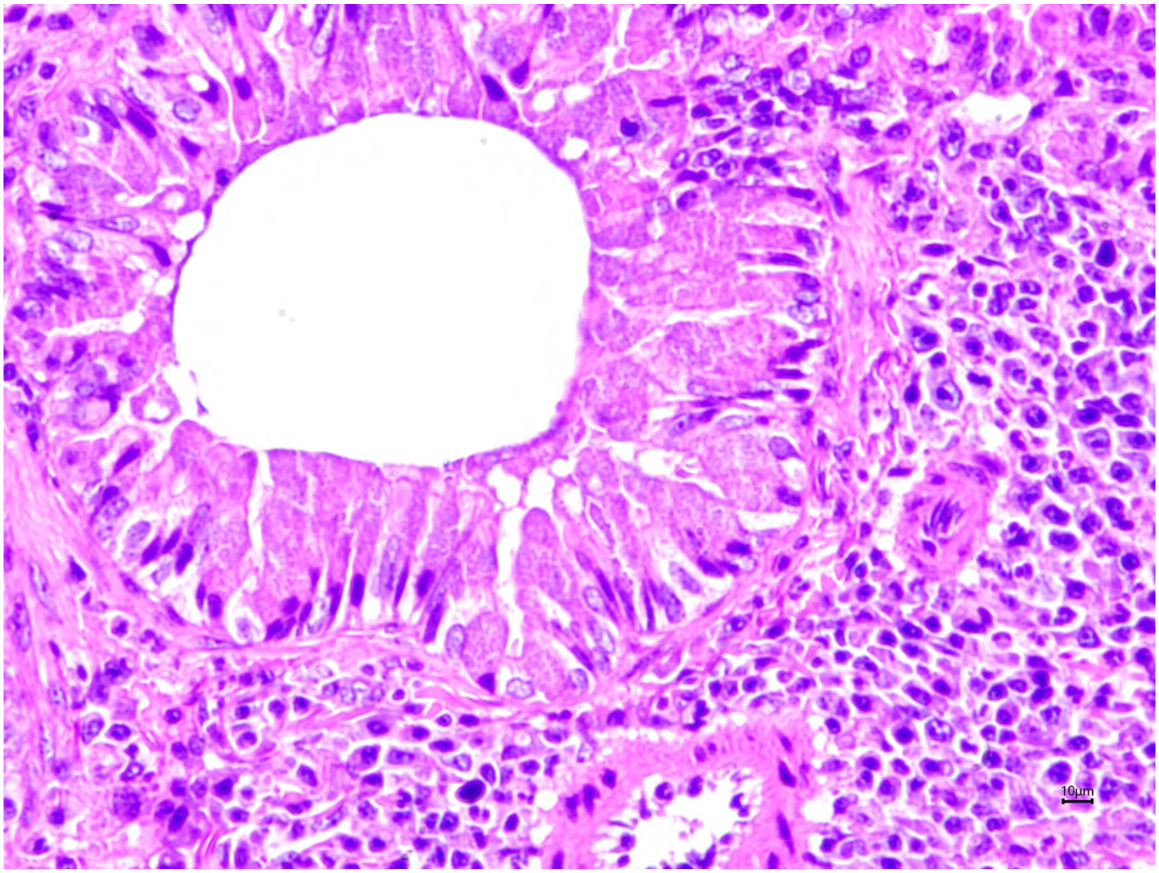

Figure 13B Control 10X

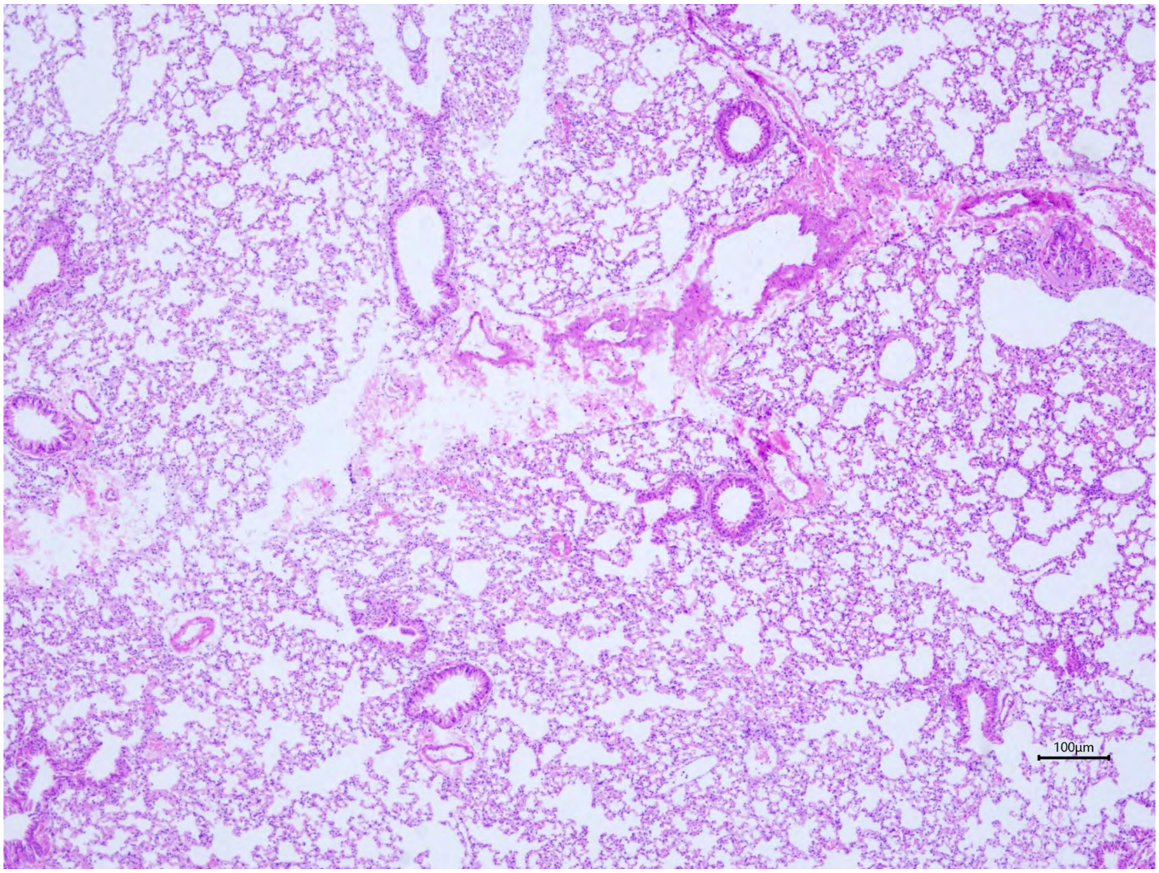

Figure 13B Control 40X

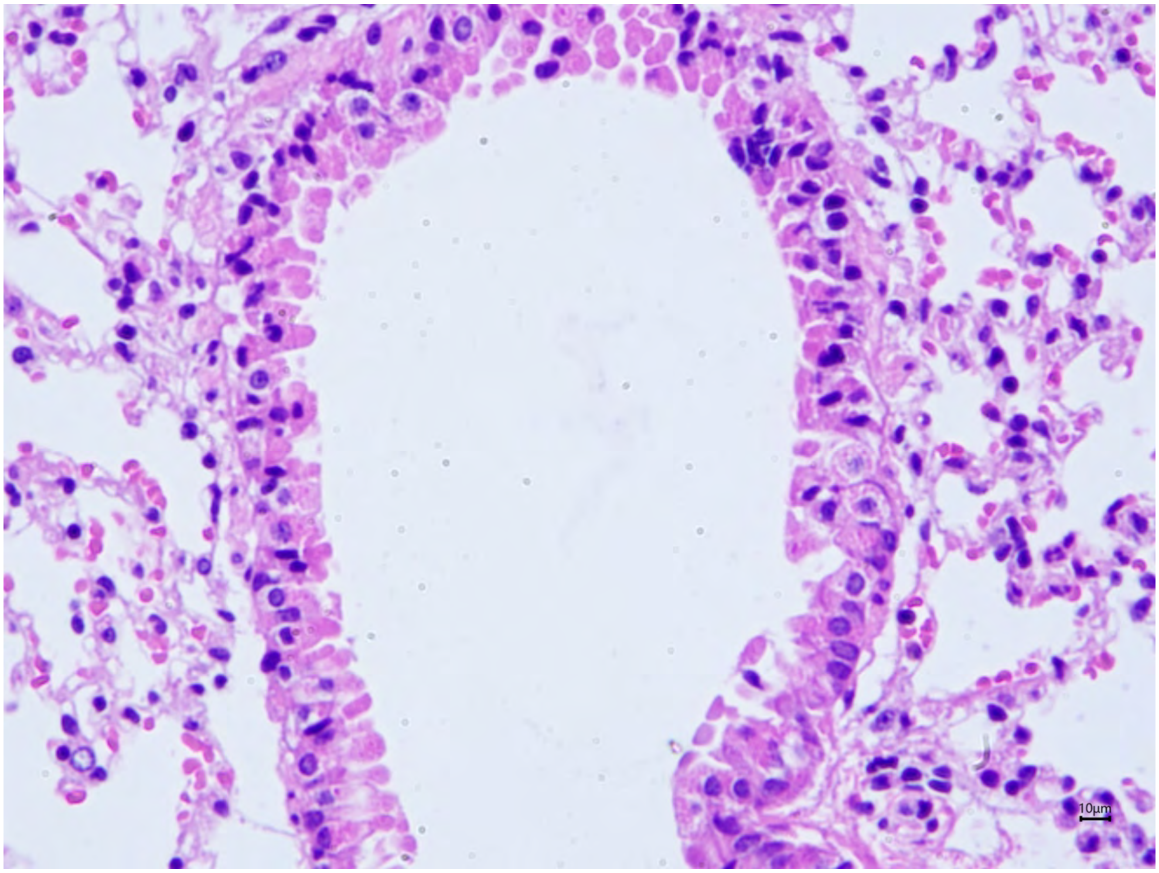

Figure 13B Diacerein 10X

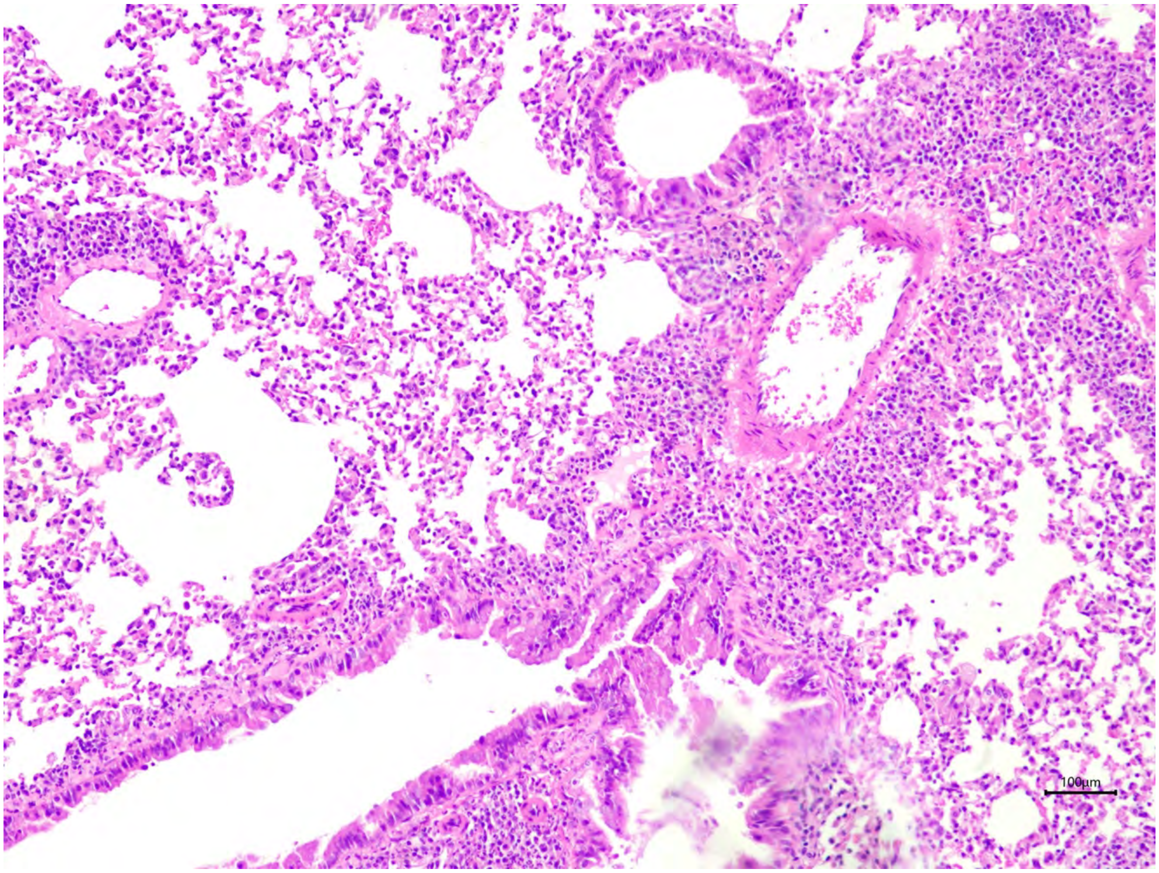

Figure 13B Diacerein 40X

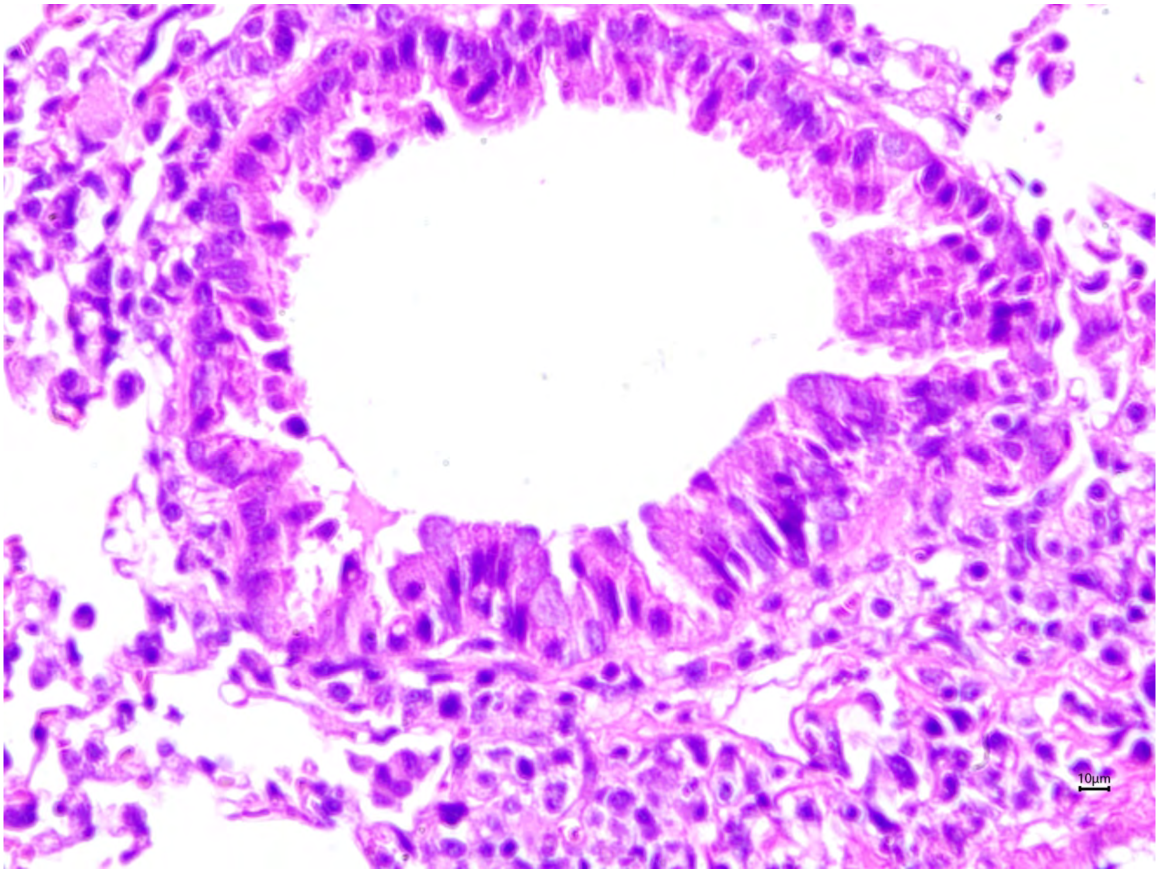

Figure 14A Asthma 10X

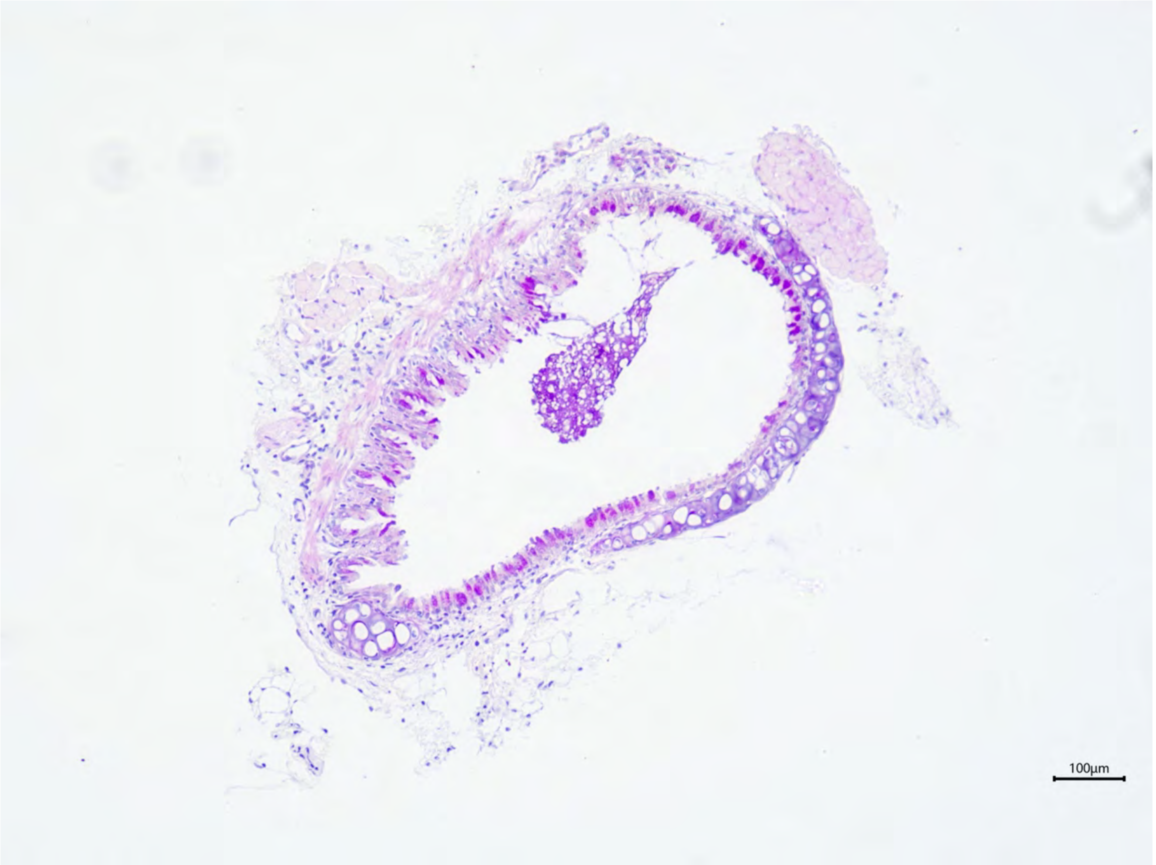

Figure 14A Asthma 40X

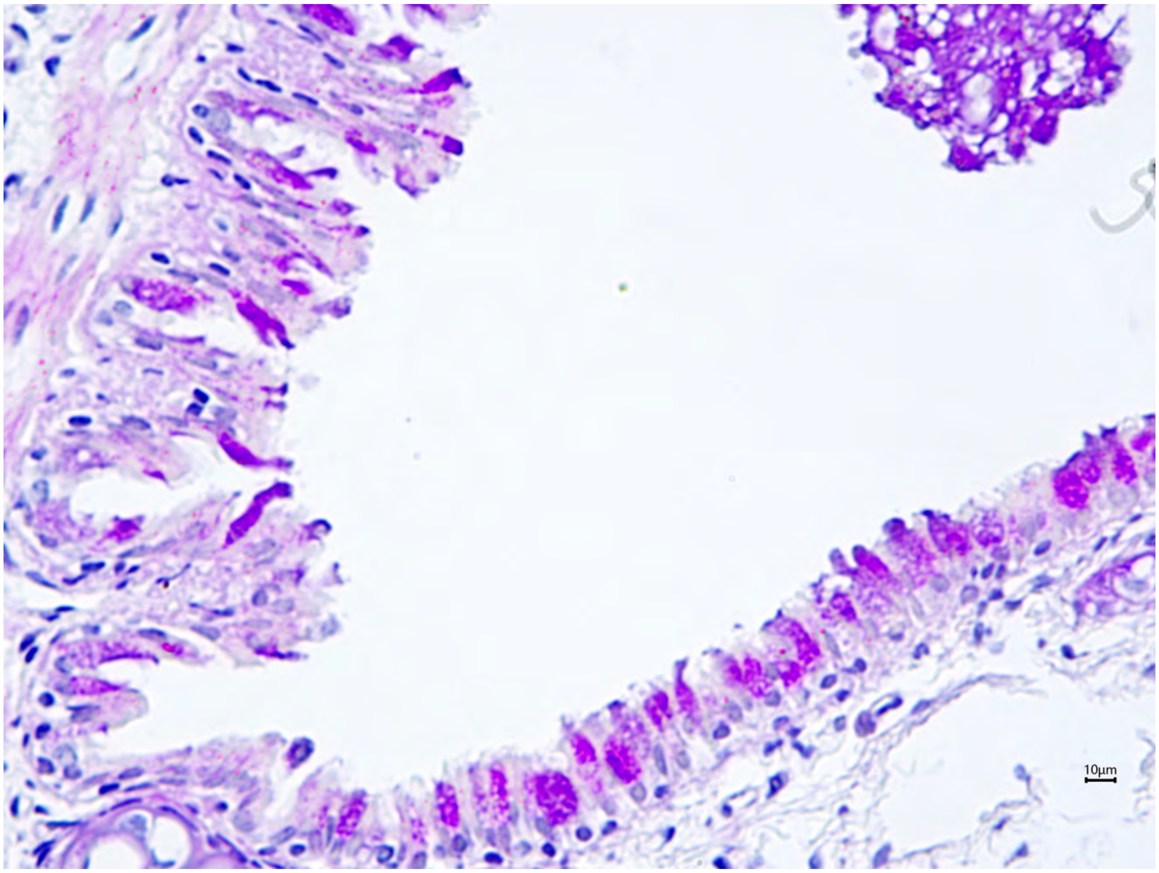

Figure 14A Control 10X

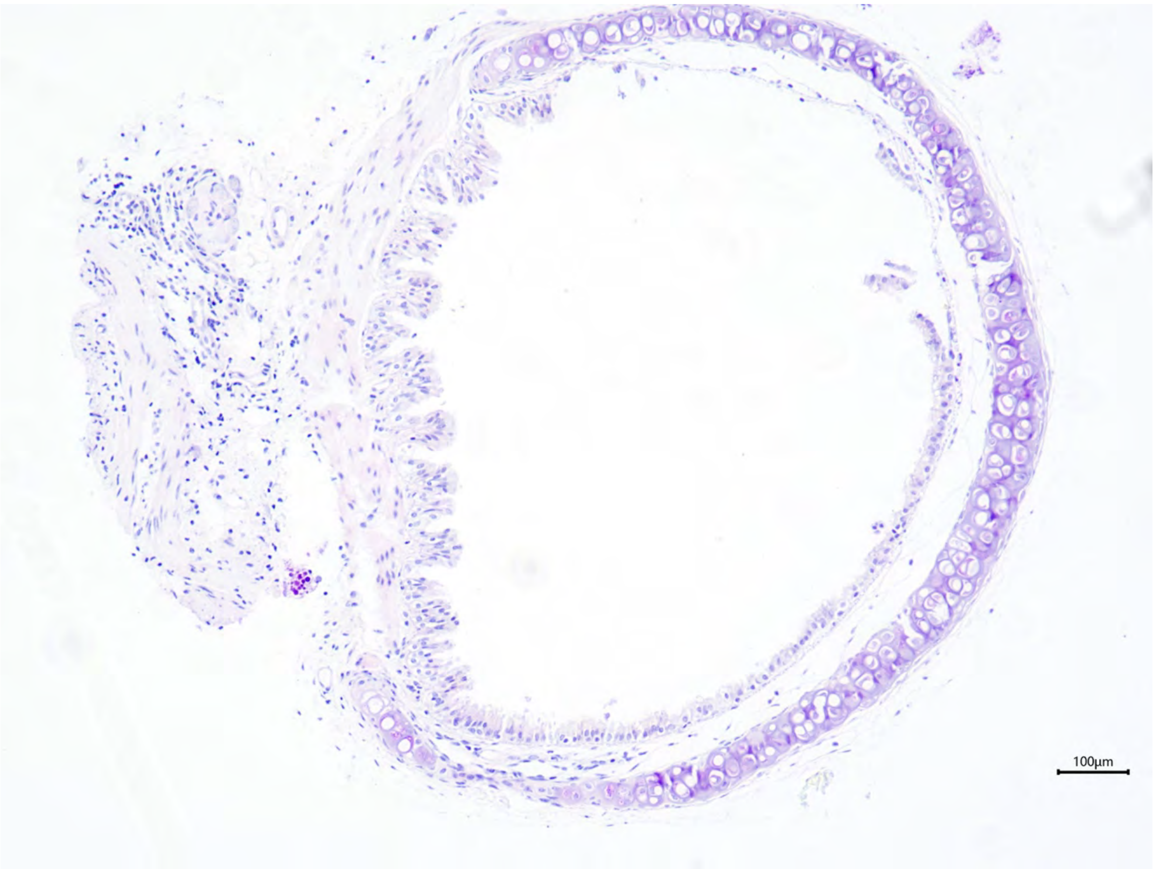

Figure 14A Control 40X

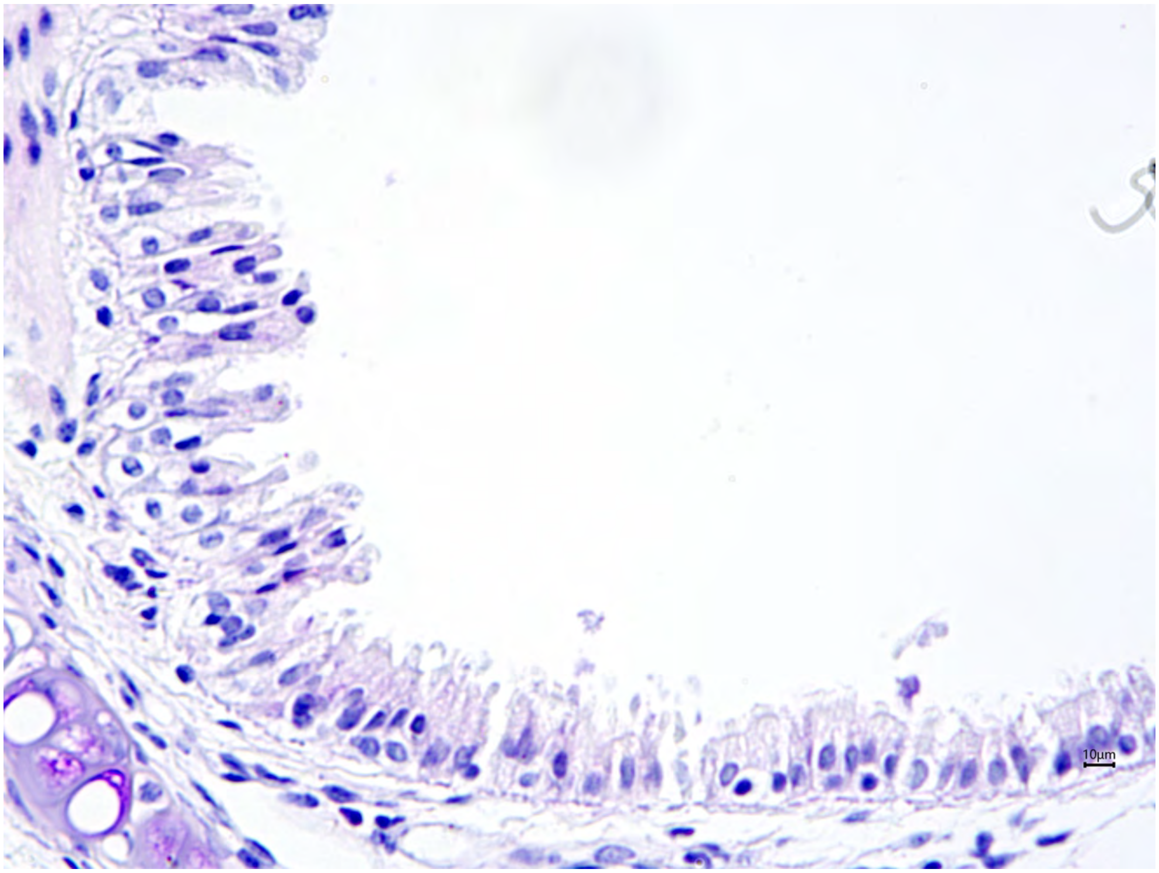

Figure 14A Diacerein 10X

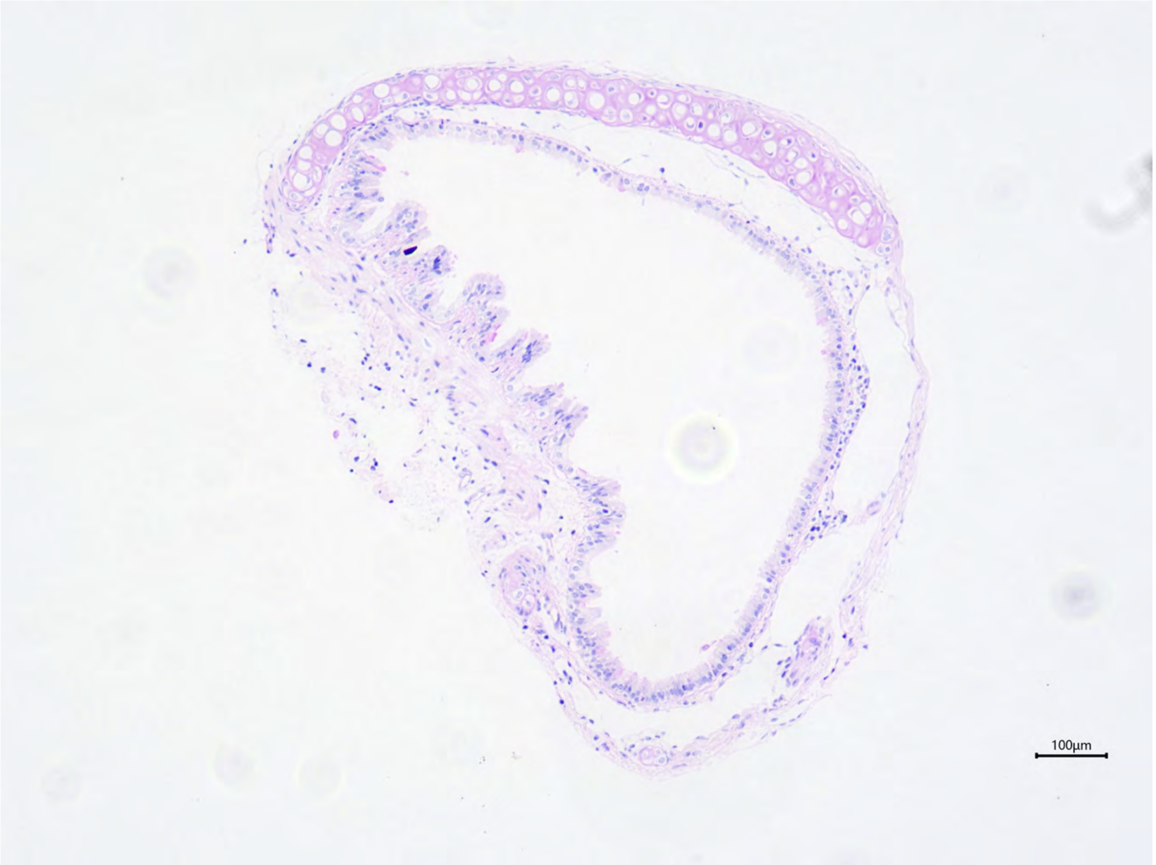

Figure 14A Diacerein 40X

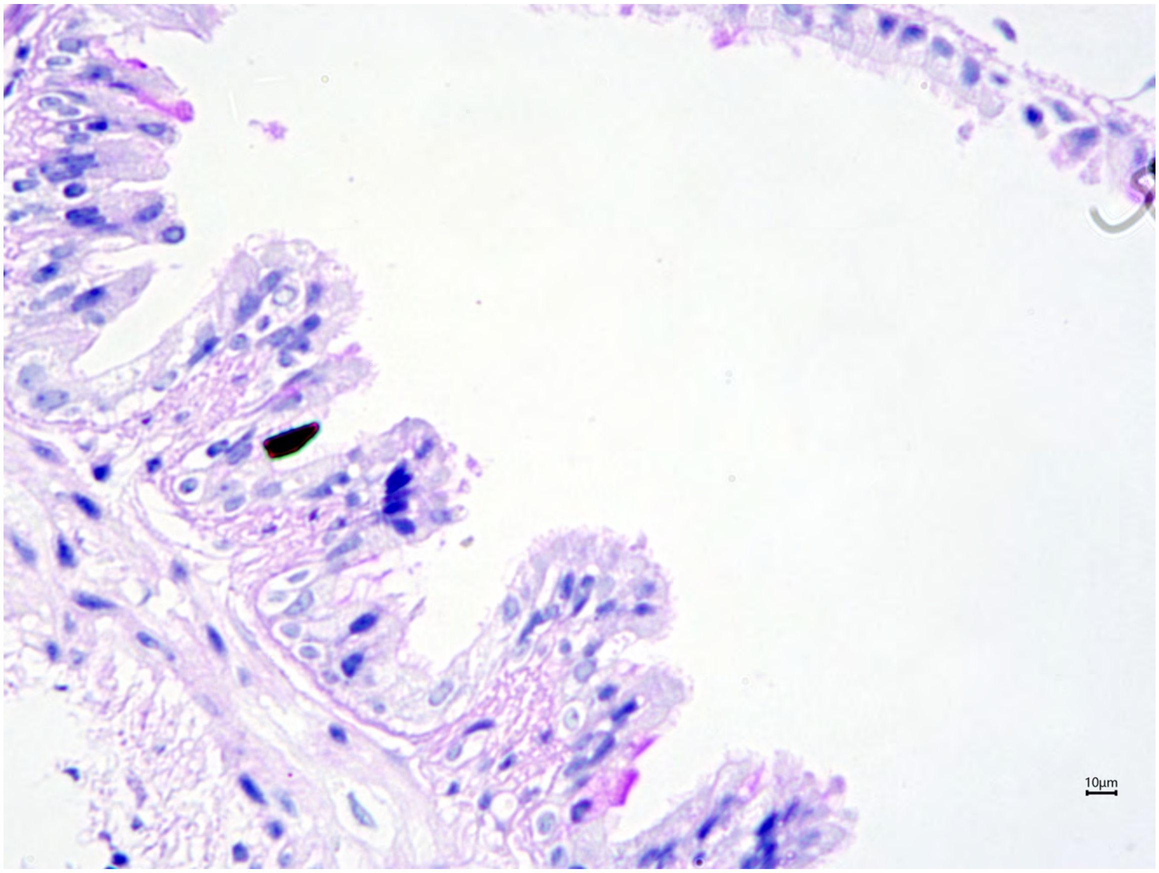

Figure 14B Asthma 10X

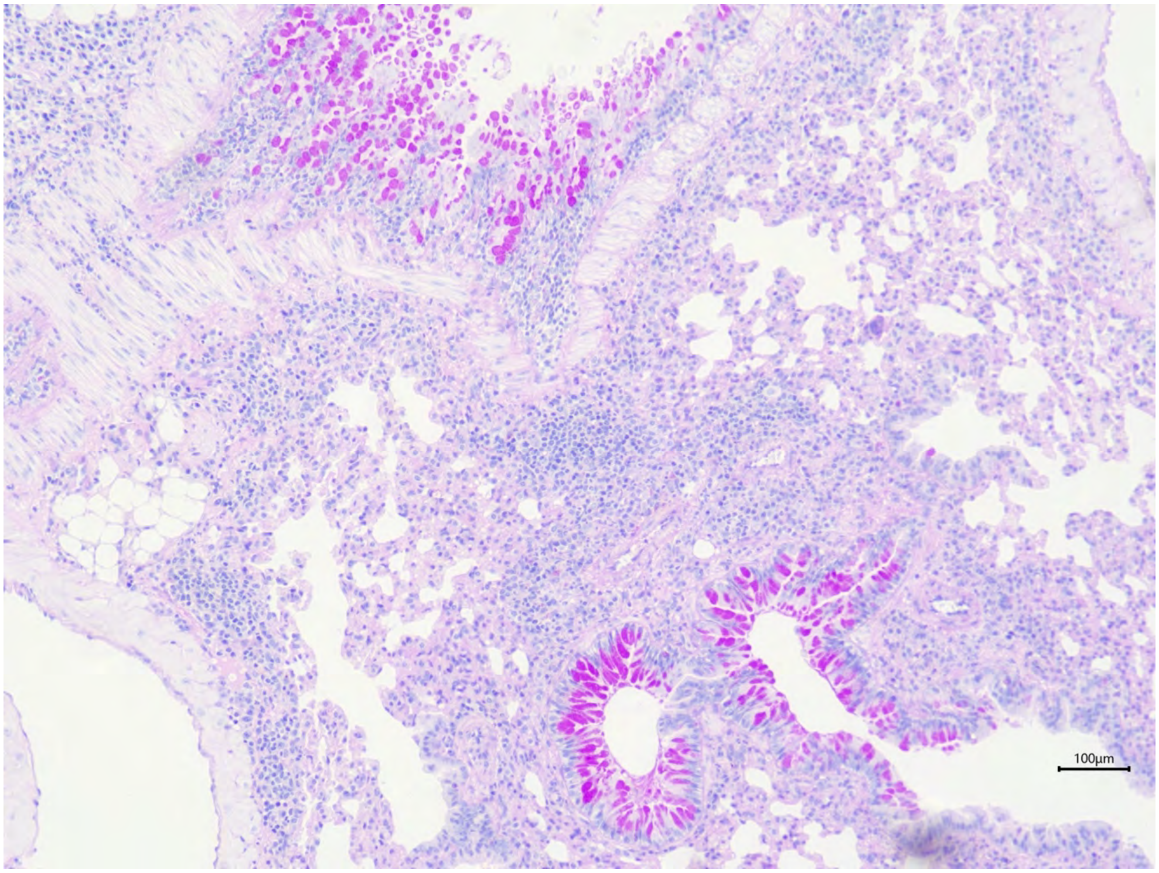

Figure 14B Asthma 40X

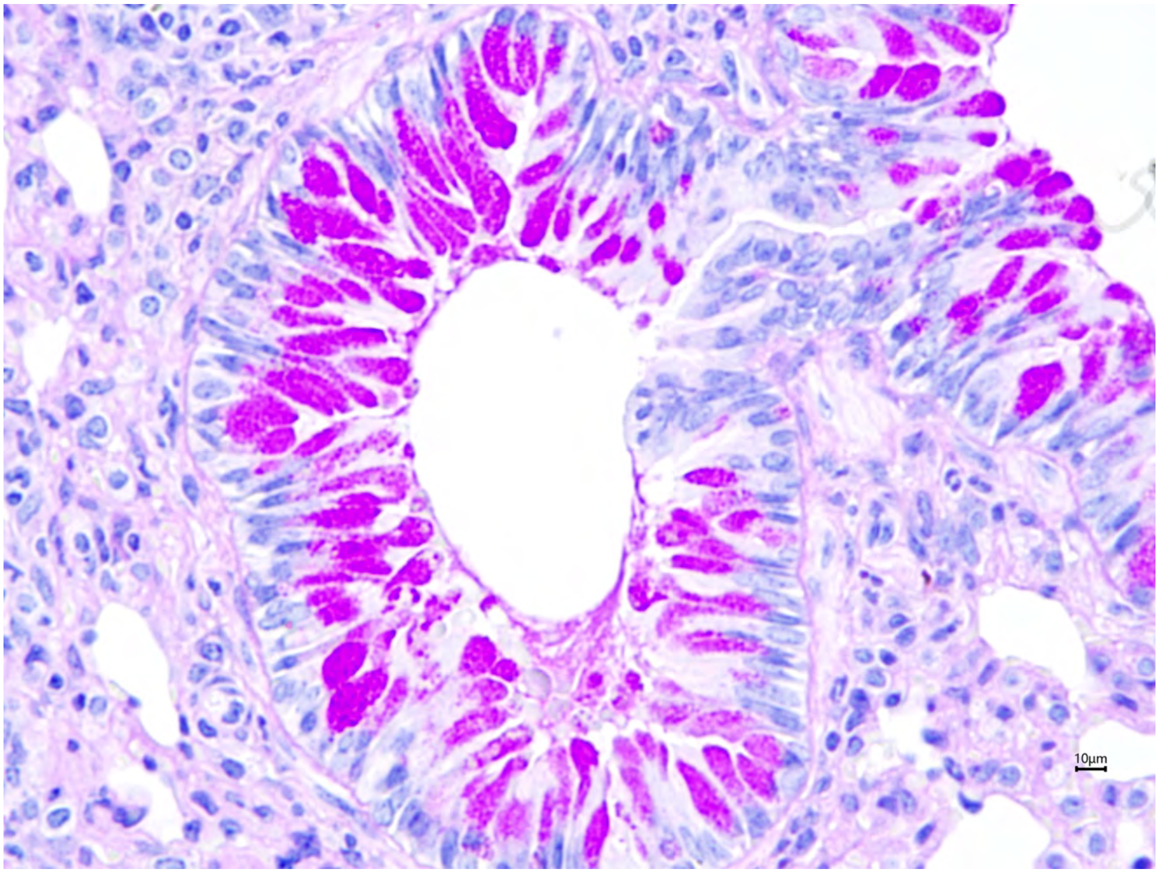

Figure 14B Control 10X

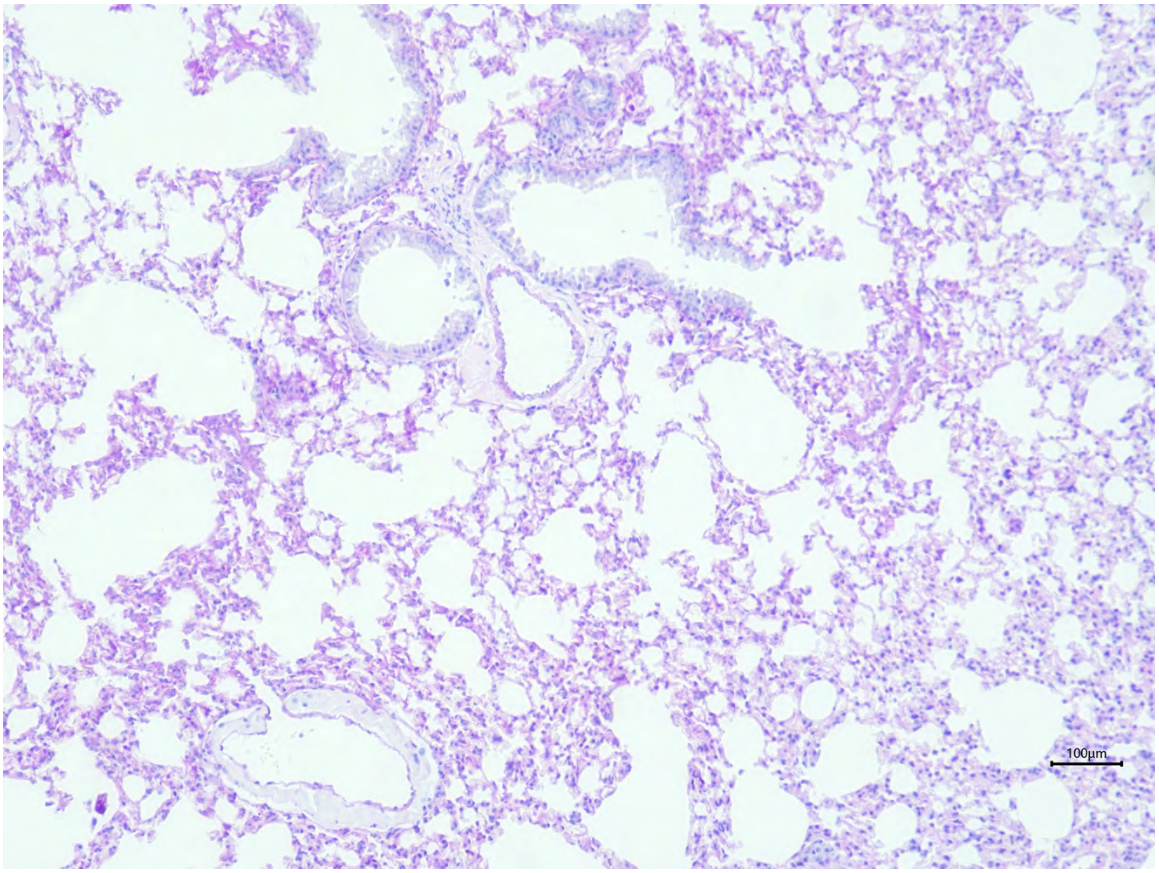

Figure 14B Control 40X

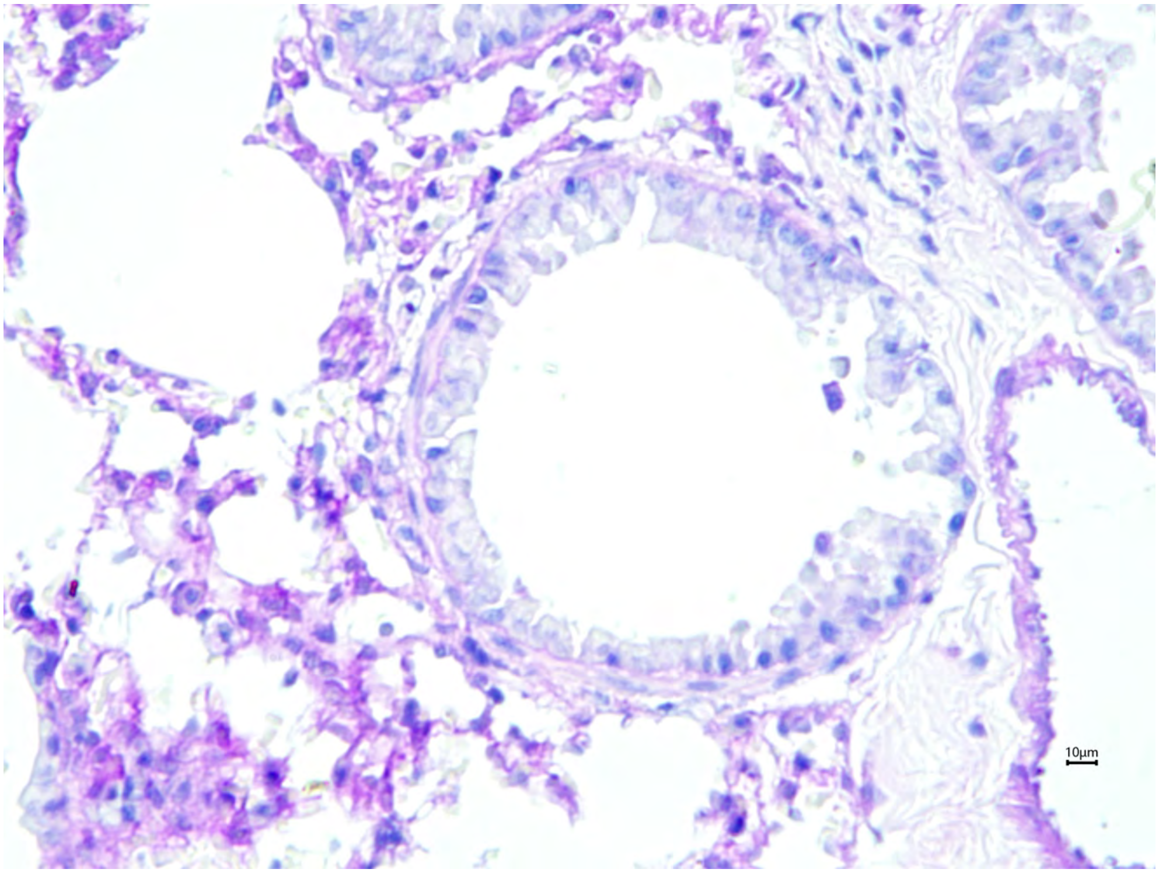

Figure 14B Diacerein 10X

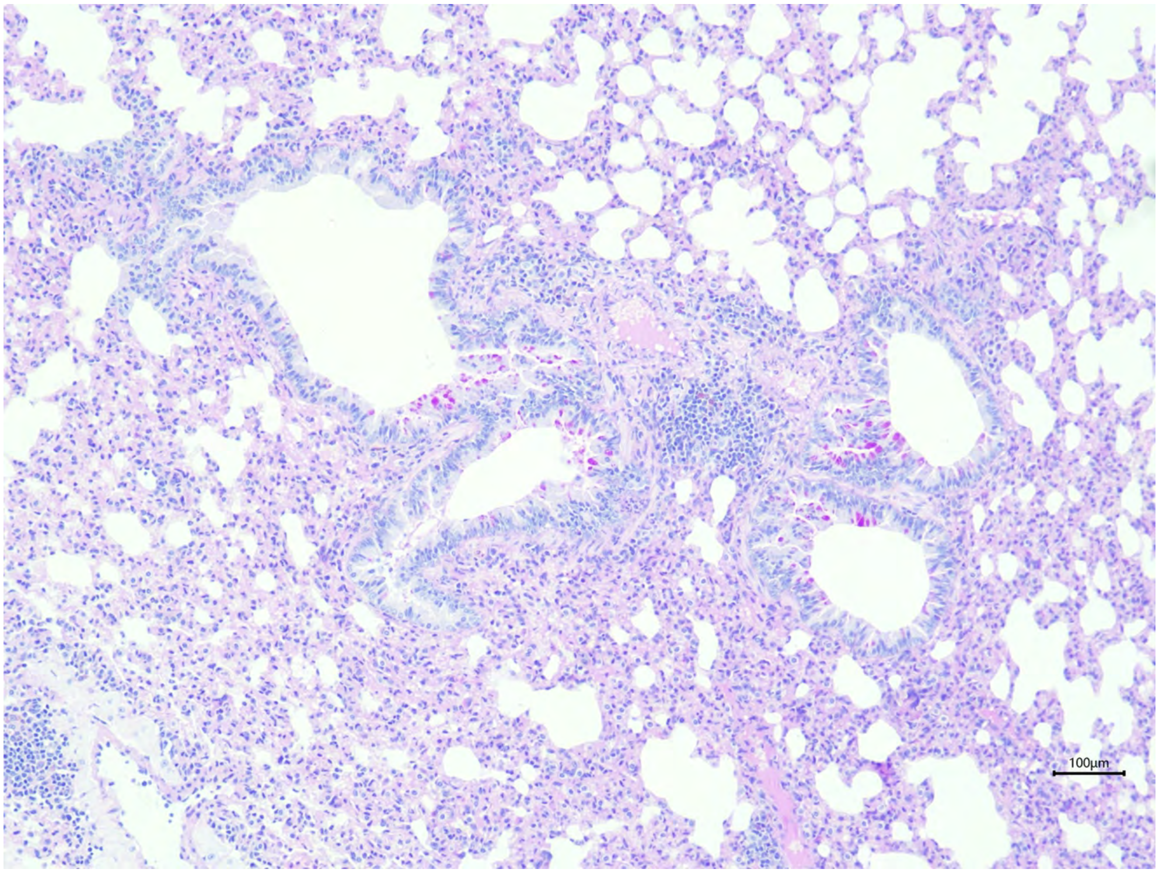

Figure 14B Diacerein 40X

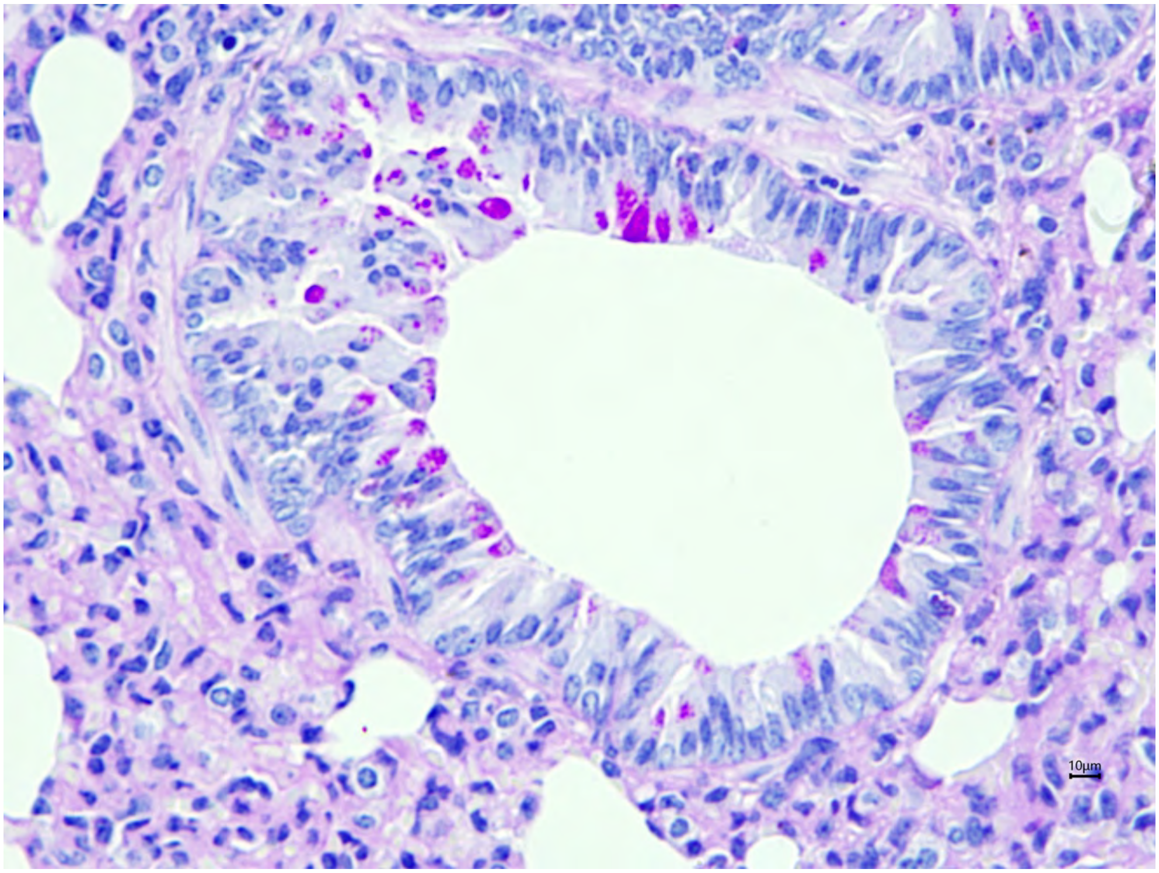

Supplement: Supplementary file 1 [file DataSheet_1.pdf]
