# Supplementary figures and images for: Anti-Contractile and Anti-Inflammatory Effects of Diacerein on Isolated Mouse Airways Smooth Muscle and Mouse Asthma Model
Source: Front Pharmacol. 2020 Sep 4;11:560361. doi: 10.3389/fphar.2020.560361 (PMC7498646; doi:10.3389/fphar.2020.560361)

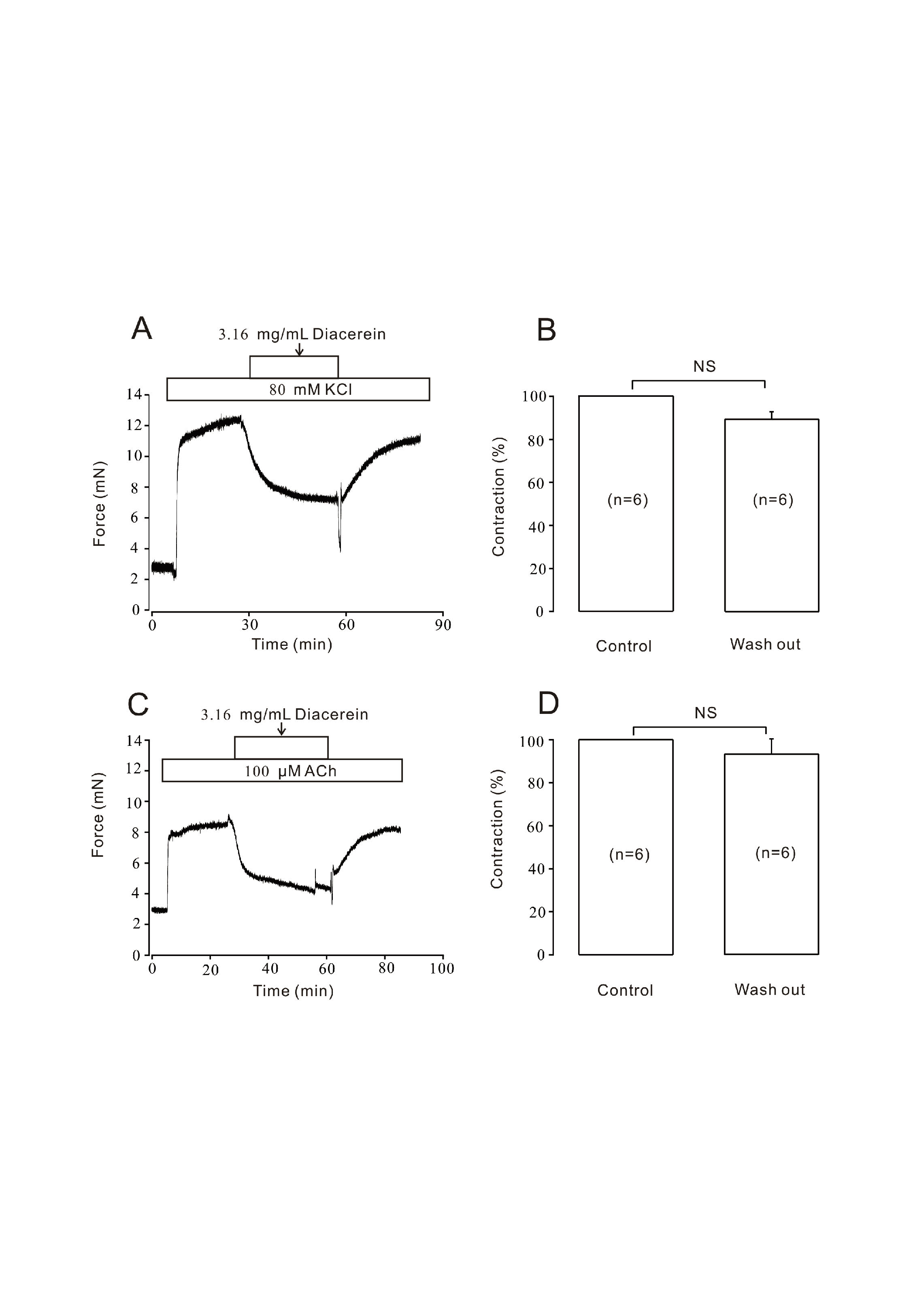

Supplement: Figure S1 — Diacerein has no harmful effect on mTRs bioactivity. (A) High K+ induced a steady-state contraction in a mTR, which was partially inhibited by 3.16 mg/mL diacerein. After washing out, a similar contraction was evoked by high K+ (n = 6/6 mice). (B) The bar graph showed no significant differences between forces at the control and wash-out stage. (C) 100 μM ACh-induced contraction could be almost completely inhibited by 3.16 mg/mL diacerein. After washing out, a similar contraction was evoked by 100 μM ACh. (D) The bar graph showed no significant differences between initial contraction and contraction after wash-out. NS, no significant. [file Image_1.jpeg]
